# Genetic fine-mapping from summary data using a nonlocal prior improves the detection of multiple causal variants

**DOI:** 10.1093/bioinformatics/btad396

**Published:** 2023-06-22

**Authors:** Ville Karhunen, Ilkka Launonen, Marjo-Riitta Järvelin, Sylvain Sebert, Mikko J Sillanpää

**Affiliations:** Research Unit of Mathematical Sciences, University of Oulu, Oulu, P.O.Box 8000, FI-90014, Finland; Research Unit of Population Health, University of Oulu, Oulu, Finland; Research Unit of Mathematical Sciences, University of Oulu, Oulu, P.O.Box 8000, FI-90014, Finland; Research Unit of Population Health, University of Oulu, Oulu, Finland; Department of Epidemiology and Biostatistics, Imperial College London, London, United Kingdom; Department of Life Sciences, College of Health and Life Sciences, Brunel University, London, United Kingdom; Research Unit of Population Health, University of Oulu, Oulu, Finland; Research Unit of Mathematical Sciences, University of Oulu, Oulu, P.O.Box 8000, FI-90014, Finland

## Abstract

**Motivation:**

Genome-wide association studies (GWAS) have been successful in identifying genomic loci associated with complex traits. Genetic fine-mapping aims to detect independent causal variants from the GWAS-identified loci, adjusting for linkage disequilibrium patterns.

**Results:**

We present “FiniMOM” (fine-mapping using a product inverse-moment prior), a novel Bayesian fine-mapping method for summarized genetic associations. For causal effects, the method uses a nonlocal inverse-moment prior, which is a natural prior distribution to model non-null effects in finite samples. A beta-binomial prior is set for the number of causal variants, with a parameterization that can be used to control for potential misspecifications in the linkage disequilibrium reference. The results of simulations studies aimed to mimic a typical GWAS on circulating protein levels show improved credible set coverage and power of the proposed method over current state-of-the-art fine-mapping method SuSiE, especially in the case of multiple causal variants within a locus.

**Availability and implementation:**

https://vkarhune.github.io/finimom/.

## 1 Introduction

Leveraging genetic associations from up to millions of individuals, genome-wide association studies (GWAS) have been widely used to find genomic loci associated with complex traits and disease liability (Visscher *et al.* 2017). Such genomic regions can be further prioritized to analyze disease etiology and potential pharmacological targets in more detail (Gallagher and Chen-Plotkin 2018).

Each genomic loci highlighted in a GWAS may harbor several causal variants [such as single-nucleotide polymorphisms (SNPs)], and the identification of these variants is important for a better understanding of the biological mechanisms underlying the trait of interest. The difficulty in dissecting the causal variants within a specific locus is compounded by the linkage disequilibrium (LD) patterns, as noncausal variants in LD with a true causal variant will also show associations with the trait of interest.

Fine-mapping methods aim to distinguish independent causal variants for a given trait within a specific genomic locus (Schaid *et al.* 2018). Assuming additive effects of individual variants, fine-mapping can be considered as a variable selection problem, where the aim is to identify the true causal signals from a candidate set of genetic variants (O’Hara and Sillanpää 2009, Fan and Lv 2010).

As the effect sizes of individual genetic variants are typically minuscule, large sample sizes are required in both GWAS and fine-mapping to obtain adequate statistical power to detect robust genetic associations. In addition to practical difficulties and privacy concerns in providing access to large-scale individual-level genetic data, summarized genetic associations from GWASs are increasingly publicly available. Therefore, the use of summary-level genomic data has become a standard in carrying out post-GWAS analyses (Pasaniuc and Price 2017). Accordingly, many of the common fine-mapping methods are either compatible with or developed for GWAS summary statistics (The Wellcome Trust Case Control Consortium et al. 2012, Yang *et al.* 2012, Hormozdiari *et al.* 2014, Kichaev *et al.* 2014, Chen *et al.* 2015, Kichaev and Pasaniuc 2015, Benner *et al.* 2016, Newcombe *et al.* 2016, Wang *et al.* 2020, Zou *et al.* 2022).

A key element in fine-mapping using summarized data is the correct specification of the LD structure underlying the locus-specific genetic associations. The most simple method of summary data fine-mapping via Bayes factors (The Wellcome Trust Case Control Consortium et al. 2012) makes a simplifying assumption of one causal variant per locus. This strategy has the benefit of not needing LD information, albeit the assumption itself may not be realistic (Abell *et al.* 2022). However, if no in-sample LD information is available, care must be taken in how to obtain the LD reference. Ideally, the reference is derived from a sufficiently sized sample (Benner *et al.* 2017) that is ancestrally similar to the population in the summary statistics, and with similar data quality control procedures applied (Kanai *et al.* 2022). In practice, this target may be very difficult to achieve (Kanai *et al.* 2022).

Here, we propose a Bayesian fine-mapping method for quantitative traits based on nonlocal product inverse-moment (piMOM) priors using summary-level genetic associations. Originally proposed by Johnson and Rossell (2010), nonlocal prior densities have zero density at the null parameter value, and the nonlocal product priors are independent products of such densities (Johnson and Rossell 2012). Such priors for regression coefficients have attractive properties of both theoretical and finite-sample performance for variable selection and prediction (Johnson and Rossell 2012, Rossell and Telesca 2017, Shin *et al.* 2018). In the context of genomic analyses, nonlocal priors for binary, continuous, and time-to-event outcomes have been developed for individual-level data (Nikooienejad *et al.* 2016, Sanyal *et al.* 2019, Nikooienejad *et al.* 2020). Prior to our work, no methods based on nonlocal priors have been applied on summary-level genetic data. Of note, analyses of summary-level genomic data provide an additional benefit of algorithms not scaling with the sample size.

The proposed method allows a flexible way to take the external LD information into account. We treat the model dimension as a parameter for which we assign a beta-binomial prior distribution. Such prior has been shown to provide optimal model selection in high-dimensional settings (Castillo *et al.* 2015). Crucially, our formulation of the prior allows the proposed method to be adjusted according to whether an in-sample or out-of-sample LD information is used.

We further apply the approximate Laplace’s method (Rossell *et al.* 2021) and a locally balanced proposal (Zanella 2020) in our model selection posterior sampling algorithm, leading to excellent computational efficiency. We demonstrate our method’s competitive performance to the current state-of-the-art fine-mapping method SuSiE (sum-of-single-effects regression) by different simulated scenarios and an applied example.

## 2 Materials and methods

### 2.1 Statistical model

Let $Y={\left(y_{1}\;\ldots \;y_{N}\right)}^{T}$ be a vector of the mean-centered observed values for a quantitative trait of interest, and ***X*** a standardized (mean = 0; standard deviation = 1) genotype matrix of size *N *×* P* for genetic variants within a specific genomic locus. Given a linear model where $\varepsilon ∼MVN_{N}(0,\text{diag}(\sigma_{\varepsilon }^{2}))$, our aim is to identify the causal variants *x_j_* for which $\beta_{j}\neq 0$. In other words, we aim to identify a model (or a causal configuration) ***m***, which only includes the *d* causal variants, from a set of candidate models M of maximum size (dimension) *K*. Typically, the magnitude of variants included in a fine-mapping application vary from $P\approx 10^{2}$ to $P\approx 10^{3}$, and it is also reasonable to assume that the true model dimension d≤K≪P.


(1) Y=Xβ+ε,

In the absence of individual-level genetic data, we resort to the use of GWAS summary statistics, where the outcome is regressed on each genetic variant separately, with population stratification adequately taken into account, and with further adjustments for additional covariates, such as sex, age or technical covariates, carried out where appropriate (Uffelmann *et al.* 2021). We require that the coefficient estimates ${\hat{\beta }}_{j}$ and their standard errors $\text{SE}({\hat{\beta }}_{j})$ are available in the GWAS summary statistics. Furthermore, we assume that there is a good estimate available for the LD matrix $R_{P\times P}$, and that the effect allele frequencies (EAFs) for each variant and Var(Y) are known.

Assuming a large *N* and small *β_j_* (both of which are usually the case in GWAS), we follow the proposal by Zhu and Stephens (2017) and write the likelihood for the observed effect estimates $\hat{\beta }$ as where $\hat{S}=\text{diag}(\text{SE}({\hat{\beta }}_{1})\;\ldots \;\text{SE}({\hat{\beta }}_{J}))$ and $\hat{R}$ is an estimate of LD matrix ***R***. If the summary statistics are given in per-allele units, these can be trivially transformed into standardized units by assuming a Hardy–Weinberg equilibrium for the variants and multiplying both ${\hat{\beta }}_{j}$ and $\text{SE}({\hat{\beta }}_{j})$ by $\sqrt{2{\text{EAF}}_{j}(1-{\text{EAF}}_{j})/\text{Var}(Y)}$.


(2) $$\pi (\hat{\beta }|\beta )=|2\pi \hat{S}\hat{R}\hat{S}{|}^{-1/2}\;\text{exp}\;\left\{-\frac{1}{2}\left({\left(\hat{\beta }-\hat{S}\hat{R}{\hat{S}}^{-1}\beta \right)}^{T}{\left(\hat{S}\hat{R}\hat{S}\right)}^{-1}(\hat{\beta }-\hat{S}\hat{R}{\hat{S}}^{-1}\beta )\right)\right\},$$

#### 2.1.1 Nonlocal prior for effect size β

A Gaussian density is commonly used for an effect size prior of a causal variant. Such zero-centered, i.e. “local,” priors have the maximum density for the causal effect size at the null parameter value. However, when conditioning on model ***m*** which only includes the causal variant(s), such prior densities may seem counter-intuitive due to unnecessary strong shrinkage of the causal variants toward the null value. Therefore, conditioned on model ***m***, we set a nonlocal product inverse-moment (piMOM) prior for the causal effect size vector β: where τ>0, r∈1,2,…, and Γ(·) is the gamma function. This prior is a product of independent inverse-moment prior densities for each component of the parameter vector in the model. As per the definition of a nonlocal prior, the density value at zero is 0, which is a natural characteristic for the effect size prior. The product formulation of the prior ensures that the density is zero if any of the components in the parameter vector is zero, which leads to a strong penalty on the parameters (Johnson and Rossell 2012). Of note, in Equation (3), we also implicitly condition on the model dimension *d*.


(3) $$\pi_{\beta }(\beta |\tau ,r,m)=\prod_{k\in m}\frac{\tau^{r/2}}{\Gamma (r/2)}|\beta_{k}{|}^{-(r+1)}\;\text{exp}\;\left(-\frac{\tau }{\beta_{k}^{2}}\right),$$

Parameter *r* controls the tail behavior of the distribution. Selecting *r *=* *1 leads to Cauchy-like tails, which are known to protect against the potential over-shrinkage of large effect sizes for sparse signals (Carvalho *et al.* 2010).

The parameter *τ* controls the spread of the effects away from 0, in that smaller *τ* allows for smaller causal effect sizes to be detected. As the magnitude of the smallest detectable effect sizes depends on the sample size, we suggest a formal way for selecting *τ* based on *N* such that $P(|\beta_{j}|>N^{-1/2}z_{q})=1-q$. For default values, we suggest $z_{q}=3.29$ and *q *=* *0.05 (Supplementary Methods and Table S1).

**Figure 1.**
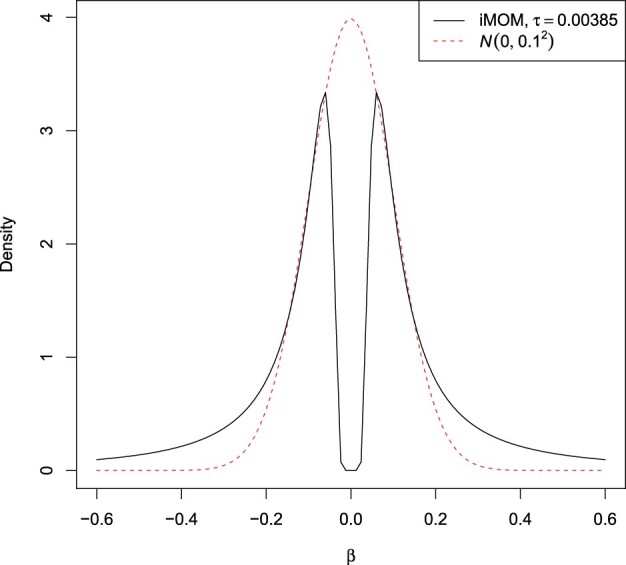
Comparison of a marginal inverse-moment (iMOM, solid black line) distribution with τ=0.00385 and a Gaussian (*N*, dashed red line) distribution with mean 0 and variance $0.1^{2}$.

Figure 1 depicts a comparison of a marginal inverse-moment density with a Gaussian density, which is commonly used as an effect size prior in genetics (Wakefield 2009). The inverse-moment prior possesses heavier tails which allow for less shrinkage of large effect sizes, and while its density vanishes as $\beta_{j}\to 0$, there is still a non-zero prior probability to detect small but non-zero effect sizes.

#### 2.1.2 Prior for model dimension and accounting for potential linkage disequilibrium misspecifications

As a prior for model dimension *d* (i.e. for the number of causal variants), we assign a beta-binomial distribution: where a,b>0,  d=1,…,K, and B(·) is the Beta function. This prior arises from a binomial distribution for the model dimension with parameters *P* and *q*, where *q* is being assigned a Beta(*a*, *b*) distribution. As fine-mapping is typically applied in situations with at least one causal variant, we set P(d=0)=0. The model dimension is also restricted by a maximum model dimension K≪P, with P(d=l)=0 for l=K+1,…,P. The beta-binomial prior is consistent with the family of priors suggested by Castillo *et al.* (2015) for recovering the correct sparse model in high-dimensional regression.


(4) $$\pi_{d}(d|P,a,b)=\left(\begin{array}{c} P\\ d \end{array}\right)\frac{B(a+d,P-d+b)}{B(a,b)},$$

One choice for the parameters *a* and *b*, as discussed by Castillo *et al.* (2015) and Castillo and van der Vaart (2012), is to set *a *=* *1 and $b=P^{u}$, *u *>* *1. The hyperparameter *u* controls the concentration of the probability mass of the model dimension prior, in that larger *u* prioritizes smaller models *a priori* (Supplementary Figure S1). Earlier work on summary data fine-mapping has shown that a misspecified LD matrix is likely to lead to more false positives (Benner *et al.* 2017). Therefore, a natural and an adaptive way to take into account the potential misspecification of the LD matrix is by increasing *u*, which places more prior probability mass to models of smaller dimension. This protects against false positives in the posterior while allowing the data to pick up strong signals of multiple causal variants, provided that the data strongly favor such models. Importantly, in the case of in-sample (or otherwise highly accurate) LD matrix being available, such strong shrinkage toward sparse models is not necessarily needed, and therefore *u* can be set to a smaller value, thus not compromising power at the expense of the false positives.

All model hyperparameters and our reasoning for choosing their values are summarized in Supplementary Table S2.

#### 2.1.3 Posterior inference

Let $\pi_{m}(D)$ denote the marginal likelihood of the data under model ***m***, again implicitly conditioning on the model dimension *d*. To obtain the posterior probability for model ***m*** of dimension *d*, given data $D=(\hat{\beta },\hat{S},\hat{R})$, we can apply Bayes’ rule and obtain where *j* refers to the dimension of model ***j***. When comparing different models, the denominator is the same for all models and can be canceled out in the algebra. To calculate $\pi_{m}(D)$, we use the approximate Laplace’s method (Rossell *et al.* 2021), based on a second-order Taylor approximation of the log-integrand: where $\beta_{m}$ refers to the parameter vector corresponding to the model ***m***, and $g_{{\tilde{\beta }}_{m}}$ and $H_{{\tilde{\beta }}_{m}}$ are the gradient and Hessian of *f*, respectively, evaluated at ${\tilde{\beta }}_{m}$. Plugging in Equations (2) and (3) into (6), dropping the constant terms with respect to the parameters, and denoting $\hat{z}={\hat{S}}^{-1}\hat{\beta }$, we have


$$P(m|D)=\frac{\pi_{d}(d)\pi_{m}(D)}{\sum_{j\in M}\pi_{d}(j)\pi_{j}(D)},$$

(5) $$\begin{array}{c} \pi_{m}(D)=\int \pi (\hat{\beta }|\beta_{m})\pi_{\beta }(\beta_{m})d\beta_{m}=\int e^{-f(\beta_{m})}d\beta_{m}\\ \approx e^{-f({\tilde{\beta }}_{m})}{\left(2\pi \right)}^{d/2}|H_{{\tilde{\beta }}_{m}}{|}^{-1/2}\;\text{exp}\;\{\frac{1}{2}g_{{\tilde{\beta }}_{m}}^{T}H_{{\tilde{\beta }}_{m}}^{-1}g_{{\tilde{\beta }}_{m}}\}, \end{array}$$

(6) $$f(\beta )=-\text{log}(\pi (\hat{\beta }|\beta ))-\text{log}(\pi_{\beta }(\beta )),$$

$$\begin{array}{c} f(\beta )\propto -d\left(\frac{r}{2}\text{log}(\tau )-\text{log}\;\Gamma (r/2)\right)+\\ \left(\frac{r+1}{2}\right)\sum_{i\leq d}\;\text{log}\;\left(\beta_{i}^{2}\right)+\sum_{i\leq d}\frac{\tau }{\beta_{i}^{2}}+\\ \frac{1}{2}\beta^{T}{\hat{S}}^{-1}\hat{R}{\hat{S}}^{-1}\beta -{\hat{z}}^{T}{\hat{S}}^{-1}\beta , \end{array}$$

$$\begin{array}{c} g_{{\tilde{\beta }}_{m}}=\frac{r+1}{{\tilde{\beta }}_{m}}-\frac{2\tau }{{\tilde{\beta }}_{m}^{3}}+\frac{1}{2}{\hat{S}}^{-1}\hat{R}{\hat{S}}^{-1}{\tilde{\beta }}_{m}-{\hat{z}}^{T}{\hat{S}}^{-1},\\ H_{{\tilde{\beta }}_{m}}={\hat{S}}^{-1}\hat{R}{\hat{S}}^{-1}+\text{diag}\left\{\frac{6\tau }{{\tilde{\beta }}_{m}^{4}}-\frac{r+1}{{\tilde{\beta }}_{m}^{2}}\right\}. \end{array}$$

Using the approximate Laplace’s method is considerably faster than the conventional Laplace’s method, where *f* needs to be optimized (Rossell *et al.* 2021). We set ${\tilde{\beta }}_{m}={\hat{R}}_{m}^{-1}{\hat{\beta }}_{m}$, in which the subscript *m* refers to the indices of model ***m***. In case of a numerically ill-conditioned ${\hat{R}}_{m}$ or $H_{{\tilde{\beta }}_{m}}$, we resort to solving $\pi_{m}(D)$ using the conventional Laplace’s method, as in Johnson and Rossell (2012) and Nikooienejad *et al.* (2016; Supplementary Methods).

#### 2.1.4 Algorithm and implementation

To generate dependent samples from the posterior distribution, we propose the following Markov chain Monte Carlo (MCMC) sampling scheme:

1. Choose initial model $m^{\text{curr}}$.
2. For $i=1,\ldots ,n_{\text{iter}}$
  - To create a proposal model $m^{\text{prop}}$, randomly select to either add, delete, or swap one of the active variables in $m^{\text{curr}}$, with the proposed model dimension $d^{\text{prop}}$ constrained at $1\leq d^{\text{prop}}\leq K$:
    1. Add variable with probability $p_{\text{add}}$ that is proportional to each variable’s squared correlation with the residuals of the current model: $p_{\text{add}}\propto {\left(X^{T}\varepsilon^{\text{curr}}\right)}^{2}$, where $X^{T}\varepsilon^{\text{curr}}\propto \hat{\beta }-R\beta^{\text{curr}}$.
    2. Delete variable with uniform probability from all active variables.
    3. Swap: select a variable to be swapped to inactive with uniform probability from all active variables in $m^{\text{curr}}$ – select the variable to be swapped to active with probability $p_{\text{swap}}$ that is proportional to the squared correlation with the variable to be swapped to inactive: $p_{\text{swap}}\propto r_{\text{swap}}^{2}$.
  - Using the proposed model $m^{\text{prop}}$, compute acceptance probability
    (7) $$a=\frac{m_{p}}{m_{p}+m_{c}},$$
    where
    (8) $$m_{p}=\pi_{d}(d^{\text{prop}})\pi_{m^{\text{prop}}}(D)q(m^{\text{curr}}|m^{\text{prop}}),$$
    (9) $$m_{c}=\pi_{d}(d^{\text{curr}})\pi_{m^{\text{curr}}}(D)q(m^{\text{prop}}|m^{\text{curr}}),$$

$d^{\text{prop}}$ and $d^{\text{curr}}$ are the dimensions of models $m^{\text{prop}}$ and $m^{\text{curr}}$, respectively, and q(m′|m) is the conditional probability of the proposal for model m′, given model *m*.
  - Sample u∼U(0,1), and if *a* > *u*, then set $m^{\text{curr}}=m^{\text{prop}}$.

In the “add”-step, we give the largest probabilities to variables that have the largest correlations with the residuals of the current model. This bears similarities to the screening method proposed by Shin *et al.* (2018). Our method allows a non-zero probability for all variables, instead of considering only the variables that correlate highly with the residuals of the current model. The probabilities required for $q(m^{\text{curr}}|m^{\text{prop}})$ in the “delete”-step can be cheaply evaluated by seeing that $X^{T}\varepsilon^{\text{curr}}-X^{T}\varepsilon^{\text{prop}}\propto R(\beta^{\text{prop}}-\beta^{\text{curr}})$, and that the number of non-zero elements in the vector $\left(\beta^{\text{prop}}-\beta^{\text{curr}}\right)$ is at most $\max(d^{\text{curr}},d^{\text{prop}})$.

In the “swap”-step, we prioritize variants with high correlations with a variant already existing in the model. This prevents the model from deviating far from an already good fit, in that the variant added to the model is likely in high LD with a variant to be replaced. This also provides a natural and efficient way to take into account the uncertainty of a true causal variant among a group of variants in very high LD (see also Supplementary Methods for further considerations of extremely high LD).

The acceptance probability in Equation (7) is also called the Barker proposal (Barker 1965), suggested for nonlocal priors in Johnson and Rossell (2012). Furthermore, Zanella (2020) showed this to be the optimal proposal regarding the mixing time in the case of binary indicators. Accordingly, by examining the trace plots for the realizations of the model dimension parameter in the simulations (Section 2.2), we found similar convergence for Markov chains of length 12 500 with a 2500 burn-in (i.e. 10 000 samples from the posterior) as for Markov chains of length 60 000 with a 10 000 burn-in (50 000 samples).

In Equations (8) and (9), the model dimension prior *π_d_* and the marginal likelihood $\pi_{m}$ refer to those in Equations (4) and (5), respectively. The interest is in whether the variants are causal or not, or in other words, whether they are included in model ***m***. Therefore, we have integrated out all other parameters (that is, the effect sizes β) in Equation (5) using the approximate Laplace’s method. The proposed sampling scheme is related to reversible jump MCMC algorithm (Green 1995), however our formulation and use of the approximate Laplace’s method avoids complicated sampling from varying-dimensional model space.

The proposed fine-mapping method “FiniMOM” (fine-mapping using a product inverse-moment prior) with the described sampling scheme is implemented in a freely available R package: https://github.com/vkarhune/finimom.

#### 2.1.5 Credible sets

We adopt the proposed approach by Lee *et al.* (2018) and Wang *et al.* (2020) and treat credible sets as the main tool for posterior inference. A credible set at level α∈(0,1) is defined as a set of variants that contain a true causal variant with probability larger than *α*.

Via credible sets, we can present the uncertainty in both (i) the number of causal variants that are supported by the data and (ii) the identification of a true causal variant from a candidate set of variants for a specific set (Supplementary Methods). We can directly use the posterior distribution of model dimension, P(d=l|D),1≤l≤K, to obtain the posterior probabilities for the supported number of signals *l*, and equivalently, the number of credible sets. The credible sets are then created at coverage *α_l_*, which represent the coverage of *α* conditioned on *l* signals. In addition to the credible sets, posterior inclusion probabilities (PIPs) for each variant ${\text{PIP}}_{j}=P(\beta_{j}\neq 0|D),j=1,\ldots ,J$, can be calculated as the proportion of the posterior samples where variant *j* is included in the model.

#### 2.1.6 Clumping variants and linkage disequilibrium consistency check

To improve the implementation of our method, we also present options for clumping extremely highly correlated variants, and a consistency check for the out-of-sample LD reference. The details of these procedures are given in Supplementary Methods.

### 2.2 Simulation studies

We conducted simulation studies to investigate the accuracy and speed of our proposed method. The genotype data used for simulations were obtained from two Finnish population-based pregnancy-birth cohorts, Northern Finland Birth Cohort 1966 (NFBC1966; *N *=* *5400; http://urn.fi/urn:nbn:fi:att:bc1e5408-980e-4a62-b899-43bec3755243) (Sabatti *et al.* 2009, University of Oulu 2022a, Nordström *et al.* 2022) and Northern Finland Birth Cohort 1986 (NFBC1986; *N *=* *3743; http://urn.fi/urn:nbn:fi:att:f5c10eef-3d25-4bd0-beb8-f2d59df95b8e) (Järvelin *et al.* 1993, University of Oulu 2022b) (Supplementary Methods). Genotype data from NFBC1966 were used for phenotype simulations to create the summary-level data, and genotype data from NFBC1986 for estimating the out-of-sample LD matrix.

We randomly selected five protein-coding genes of varying size from different chromosomes, and selected the region ±100 kb around each gene for fine-mapping. The variants were filtered for availability in both NFBC1966 and NFBC1986 datasets, resulting in the number of variants in the fine-mapped loci varying from 387 to 2996 (Supplementary Table S3).

We considered scenarios of 1, 2, or 5 causal variants explaining 0.015 or 0.03 of the phenotypic variance (Supplementary Methods). The parameters used in the simulations represent plausible scenarios for protein quantitative trait loci (pQTL) analyses (Ferkingstad *et al.* 2021). The resulting summary statistics (i.e. association estimates and their standard errors) and an LD matrix (see below) were given as inputs to the considered fine-mapping methods.

To investigate the influence of the choice of the LD matrix $\hat{R}$, we compared the performance of using in-sample LD matrix (i.e. calculated using NFBC1966 genotype data) and out-of-sample LD matrix, calculated using NFBC1986 genotype data. We clumped the variants at $r^{2}=0.99$, and applied the LD inconsistency check when out-of-sample LD matrix was used.

Each scenario was repeated 100 times. For all simulations, *r *=* *1, and the maximum number of causal variants *K *=* *10. We varied the hyperparameters τ∈{0.00320,0.00385,0.00538,0.0083} and u∈{1.05,1.25,1.5,1.75,2,2.25,2.5} (see Supplementary Methods and Table S1 for how *τ* values for the simulations were selected). MCMC was run for 12 500 iterations, of which we excluded the first 2500 as a “burn-in,” and used the last 10 000 for posterior inference.

The two main performance measures were the 95% credible set coverage (the proportion of the credible sets containing a true causal variant) and power (the proportion of true causal variants included in a credible set). We also evaluated the median size of the credible sets, the sets being ideally as small as possible with adequate coverage and power. The sampling variability of the estimates in the replication results is quantified by 95% confidence intervals, calculated using binomial distribution for coverage and power, and by 1000 bootstrap samples for median model dimension.

We compare our method with the current state-of-the-art method of sum-of-single-effects (SuSiE) regression using summary statistics, which has been shown to perform well against many other fine-mapping methods (Wang *et al.* 2020, Zou *et al.* 2022). The SuSiE method applies a “sum-of-single-effects” prior:


$$\begin{array}{c} \beta |b_{1},\ldots ,b_{L}=\sum_{l=1}^{L}b_{l},\\ b_{l}|\gamma_{l},b_{l}=\gamma_{l}b_{l},\;l=1,\ldots ,L,\\ \gamma_{l}∼\text{Multinomial}(1,\pi ),\\ b_{l}∼N(0,\sigma_{l}^{2}). \end{array}$$

The overall vector of effect sizes β is a sum of *L* effect size vectors with exactly one non-zero element, with a Gaussian prior for the non-zero effect size. The model fitting is done via Iterative Bayesian Stepwise Selection (IBSS) described by Wang *et al.* (2020) and Zou *et al.* (2022) extended its adaptation for summarized data. We applied SuSiE with *L *=* *10 and with the effect size prior variance $\sigma_{l}^{2}$ estimated from the data. The independent credible sets (at most *L*), and variant-specific PIPs were extracted from SuSiE output.

To compare the running times of the methods and to assess the scenarios of a larger sample size and a smaller phenotypic variance explained, we conducted additional simulations using 50 000 observations from a synthetic HAPNEST genotype dataset (Wharrie *et al.* 2022; Supplementary Methods).

### 2.3 Applied real data example

We illustrate the performance of our method in real data for genetic associations of interleukin-18 (IL18), a proinflammatory cytokine that stimulates several cell types as an inflammatory factor (Akdis *et al.* 2011). The source GWAS summary statistics on IL18 are based on the analysis of 3675 individuals in three Finnish cohorts (Ahola-Olli *et al.* 2017, Kalaoja *et al.* 2021), which identified three loci with at least one variant associated with circulating IL18 levels at $p<5\times 10^{-8}$. These three loci (±1 Mb from the variant with the lowest *P*-value) were selected for fine-mapping, in which we applied FiniMOM with τ=0.00566 (estimated based on the sample size), r=1,u=2.25 and *K *=* *10, and SuSiE (version 0.12.16) with *L *=* *10 and $\sigma_{l}^{2}$ estimated from the data. NFBC1966 genotype data were used to generate the (out-of-sample) LD matrix. We compare the variants contained in the credible sets given by each method, and further assess the marginal associations of these variants in an external GWAS on IL18, conducted in up to 19 195 individuals of European ancestries (Folkersen *et al.* 2020).

The analyses were conducted using R software. The scripts for simulations are available at: https://github.com/vkarhune/finimomSimulations.

## 3 Results

### 3.1 Analysis of simulation replicates

**Figure 2.**
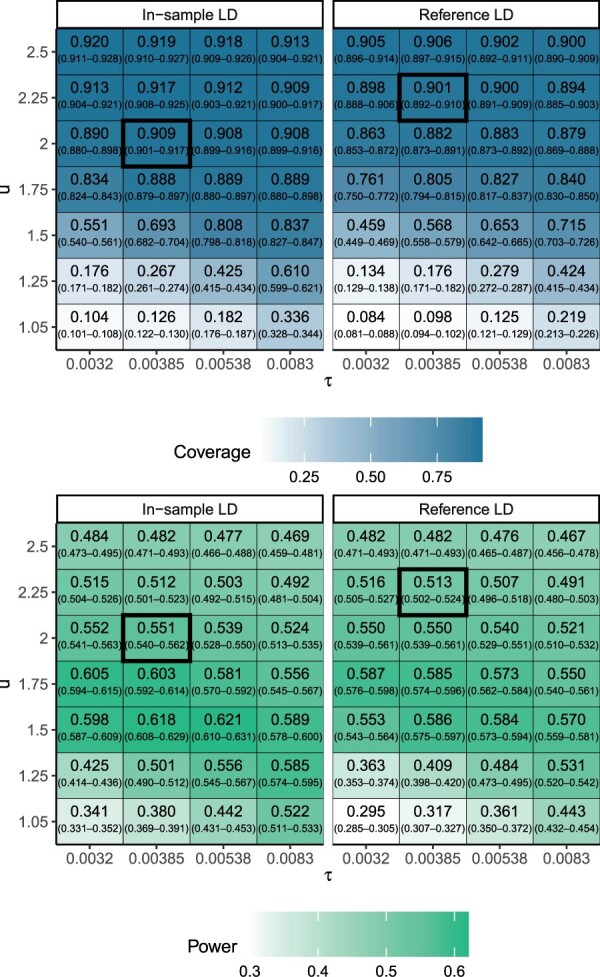
95% credible set coverage (upper panel) and power (lower panel) and their 95% confidence intervals in the Northern Finland Birth Cohort 1966 simulation study (calculated over 100 simulation replicates) using FiniMOM with different values for hyperparameters *τ* and *u*. *τ* controls the spread of the detectable effect sizes, and *u* controls the prior for model dimension. Larger values of *τ* correspond to larger causal effect sizes that can be detected, while larger values for *u* refer to stronger priors toward smaller dimensions. The parameter value combinations used in the subsequent comparisons with SuSiE are highlighted with a box. LD: linkage disequilibrium; SuSiE: sum-of-single-effects.

The FiniMOM main simulation results across all scenarios with varying values of hyperparameters *τ* and *u* are presented in Figure 2. The credible set coverage improved with larger values of *u* and smaller values of *τ*. While the credible set power also improved with decreasing *τ*, it plateaued for *u* such that the largest power was detected for *u *=* *1.5, with a slow decrease in power for larger values. As expected, using the in-sample LD consistently outperformed the use of an LD matrix from a reference panel.

Based on the investigation of FiniMOM performance using different values of *τ* and *u*, we carried out the comparison with SuSiE fine-mapping using τ=0.00385 and *u *=* *2 for in-sample LD and *u *=* *2.25 for out-of-sample LD. These values were selected as a compromise of the ideal credible set coverage and power.

**Figure 3.**
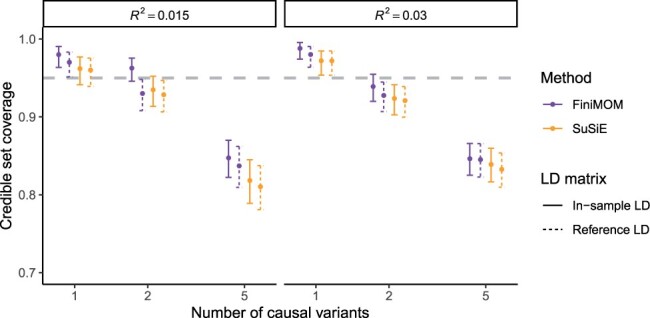
Comparison of credible set coverage and their 95% confidence intervals for FiniMOM and SuSiE in the simulation study (calculated over 100 simulation replicates). The grey dashed line represents the nominal 95% target coverage. LD: linkage disequilibrium; FiniMOM: Fine-mapping using inverse-moment priors; SuSiE: sum of single effects.

The comparison of credible set coverage between FiniMOM and SuSiE in the main simulations is shown in Figure 3. While the differences within each scenario were mostly minor, FiniMOM produced consistently larger credible set coverage than SuSiE. Interestingly, FiniMOM with out-of-sample LD was not notably worse than SuSiE with in-sample LD in any of the scenarios. SuSiE credible set coverage was superior to FiniMOM in the simulations of larger sample size (*N *=* *50, 000; Supplementary Figures S2 and S3).

**Figure 4.**
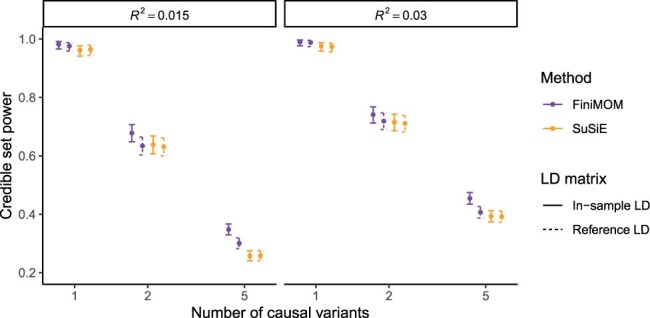
Comparison of credible set power and their 95% confidence intervals for FiniMOM and SuSiE in the simulation study (calculated over 100 simulation replicates). LD: linkage disequilibrium; FiniMOM: fine-mapping using inverse-moment priors; SuSiE: sum of single effects.

FiniMOM showed better statistical power to detect multiple causal variants than SuSiE (Figure 4). The largest differences in favor of FiniMOM were detected in the five-causal-variant scenarios. FiniMOM also outperformed SuSiE with respect to power across different sample sizes and phenotypic variance explained (Supplementary Figures S2 and S3).

The median credible set sizes were similar or somewhat larger in FiniMOM (Supplementary Figure S4). The credible set sizes differed the most in the cases of low heritability and a large number of causal variants.

When comparing the computational times, both FiniMOM and SuSiE scaled well with increasing number of variants considered (Supplementary Figure S5). Both methods also provided similar distributions for the PIPs and their ranks of simulated causal variants (Supplementary Figure S6), as well as for the number of credible sets (Supplementary Figure S7).

The coverage and power estimates across all simulation scenarios using NFBC1966 genotype data are given in Table 1. FiniMOM had better coverage and power for both in-sample LD and out-of-sample LD than SuSiE, with somewhat larger median credible set size.

**Table 1.** Estimates and 95% confidence intervals (CIs) for mean credible set coverage, mean credible set power, and median credible set size across all simulated scenarios using Northern Finland Birth Cohort 1966 genotype dataset.

Method	LD matrix	Coverage	Power	Median size
FiniMOM	In-sample	0.909 (0.901–0.917)	0.551 (0.540–0.562)	13 (13–14)
FiniMOM	Out-of-sample	0.901 (0.892–0.910)	0.513 (0.502–0.524)	12 (11–13)
SuSiE	In-sample	0.895 (0.885–0.903)	0.493 (0.482–0.504)	11 (10–12)
SuSiE	Out-of-sample	0.890 (0.880–0.899)	0.492 (0.481–0.503)	11 (10–12)

The CIs are based on binomial distribution for coverage and power, and 1000 bootstrap samples for median size.

### 3.2 Applied example

We then ran an applied example using GWAS summary statistics on circulating IL18 levels (Kalaoja *et al.* 2021). We chose three genomic regions—*NLRC4*, *RAD17*, and *BCO2—*harboring variants at $p<5\times 10^{-8}$ (± 1 Mb window from the lead variant) for fine-mapping (Table 2), and investigated the variants in the produced credible sets in a separate GWAS on IL18 levels (Folkersen *et al.* 2020) for replication (“replication GWAS”). The run times (using Intel Xeon processor running at 2.1 GHz) for *NLRC4*, *RAD17*, and *BCO2* loci were 8.3, 3.4, and 6.8 s, respectively. For SuSiE, the same run times were 5.8, 3.1, and 5.8 s, respectively.

**Table 2.** Genomic regions used in the applied example.

Lead variant	Nearest gene	Genomic region (hg19)	Variants
rs385076	*NLRC4*	chr2:31,489,851–33,489,851	6511
rs17229943	*RAD17*	chr5:67,682,536-69,682,536	3043
rs12420140	*BCO2*	chr11:111,071,294–113,071,294	5862

**Figure 5.**
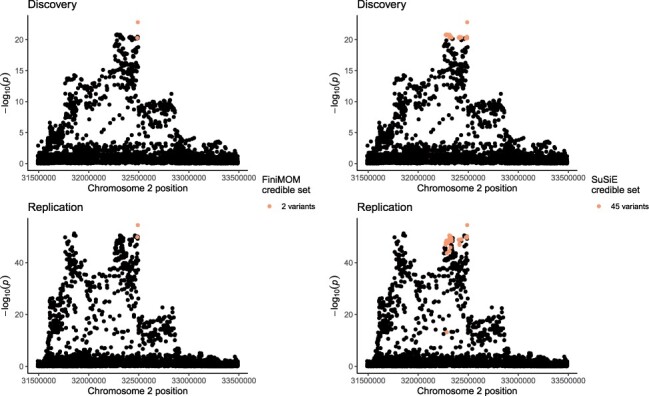
Locus plot of genetic associations ($-{\;\text{log}\;}_{10}(p)$) per each variant within NLRC4 locus (±1 Mb from rs385076 variant), with credible sets highlighted for FiniMOM (left panels) and SuSiE (right panels) in the discovery GWAS (top panels) and in the replication GWAS (bottom panels). FiniMOM: fine-mapping using inverse-moment prior; SuSiE: sum of single effects; GWAS: genome-wide association study.

The results for IL18 are summarized in Figure 5 and Supplementary Figures S8 and S9. Both methods selected the same number of credible sets for each loci, with minor deviations in the numbers of causal variants. The uncertainty in the number of credible sets is provided only by FiniMOM method (Table 3).

**Table 3.** FiniMOM posterior distribution for the number of credible sets in each locus.

	Number of credible sets				
Locus	1	2	3	4	>5
rs385076	>0.99	0.005	0	0	0
rs17229943	0.004	0.05	0.91	0.03	0
rs12420140	0.99	0.01	0	0	0

For *NLRC4*, FiniMOM produced a smaller credible set, consisting of only two variants, rs385076 and rs659239. The former SNP was also the top variant in the replication GWAS. The size of the corresponding SuSiE credible set was 45 variants (Figure 5). This is due to FiniMOM assigning larger PIP for the lead variant rs385076 (FiniMOM PIP = 0.94, SuSiE PIP = 0.70; Supplementary Figure S10). Both methods detect variant rs17229943 as the lead signal (and as its own credible set) at *RAD17* locus. In addition, there were two other credible sets detected by both methods. However, no strong signal was detected for the variants in these credible sets in the replication GWAS (Supplementary Figure S8). Both FiniMOM and SuSiE were able to distinguish the peak *BCO2*, and both produced one credible set with the same variants included. This signal was also detected in the replication GWAS (Supplementary Figure S9).

## 4 Discussion

In this article, we have proposed a novel fine-mapping method for summarized genetic associations using product inverse-moment priors. Additionally, incorporating theoretical results of the model dimension prior in very sparse regression settings, we propose an adjustable beta-binomial prior to be used. The proposed approach showed improved detection of causal variants across various scenarios which were aimed to mimic typical settings in pQTL association analyses (Ferkingstad *et al.* 2021). Our method also worked well in the applied example, is competitive to SuSiE in absolute running times, and scales well even for large genetic regions.

In Bayesian fine-mapping methods, a common way is to set a Gaussian prior for the causal effect sizes. This allows for effects that are arbitrarily close to zero with non-negligible probability (Figure 1). While such small effect sizes may exist, these cannot be reliably detected with finite datasets. In contrast, the piMOM prior applied here can be set such that, conditioned on the variant being causal, the probability for the absolute values of the detectable effect sizes being smaller than a specific threshold can be determined *a priori*. Apart from the use of nonlocal priors (Nikooienejad *et al.* 2016, Sanyal *et al.* 2019, Nikooienejad *et al.* 2020), similar approaches can be found in QTL mapping literature (Knürr *et al.* 2011, 2013, Walters *et al.* 2019), and we believe using such priors is a natural way to analyze genomic datasets.

The better performance of FiniMOM over SuSiE seems to come at the expense of slightly larger credible sets. However, based on the simulations, the differences in the set sizes were notable in the cases of low phenotypic variance explained and multiple causal variants, implying that FiniMOM better allows for uncertainty in these situations. In the scenarios of a single causal variant, the sizes were largely similar.

We also highlight our method’s flexibility in the tradeoff between credible set coverage and power, easily incorporated via the hyperparameters *τ* and *u* which control the detectable effect sizes and the model dimension prior, respectively. Similarly, FiniMOM is highly adaptable to deal with either in-sample or out-of-sample LD reference. Moreover, unlike in SuSiE, but similarly as in FINEMAP method (Benner *et al.* 2016), we also obtain the uncertainty in the number of credible sets in a fully Bayesian way.

Some limitations of our work should be mentioned. In the absence of in-sample LD information, the importance of a good LD reference cannot be overstated and, while our method is shown to be robust for out-of-sample LD references, it naturally cannot recover poor LD reference and data quality. We set the hyperparameters *r* and *a* as constants and did not conduct an exhaustive search over their parameter spaces. We did not consider the situations where the effect sizes depend on the allele frequencies of the variants (Schoech *et al.* 2019), however this can be easily incorporated by allowing the prior parameter *τ* to vary across variants (Johnson and Rossell 2012). Finally, our current method does not cover varying LD structures across different ancestries (Lu *et al.* 2022), infinitesimal polygenic effects (Cui *et al.* 2022), or multivariate outcomes (Arvanitis *et al.* 2022).

In summary, we have proposed a novel genetic fine-mapping method for summarized data that outperforms a current state-of-the-art fine-mapping method. The specific strengths in detecting multiple causal variants and adaptability to deal with out-of-sample LD information make FiniMOM an attractive option for fine-mapping studies of quantitative traits.

## Supplementary Material

btad396_Supplementary_Data

## Data Availability

NFBC1966 and NFBC1986 genotype data are available by application via http://oulu.fi/nfbc/. HAPNEST synthetic genotype data are available at: https://www.ebi.ac.uk/biostudies/studies/S-BSST936. IL18 GWAS summary statistics used in the applied example are available at: https://doi.org/10.5523/bris.3g3i5smgghp0s2uvm1doflkx9x (discovery) and https://doi.org/10.5281/zenodo.2615265 (replication).
