# Supplementary material for: Genetic fine-mapping from summary data using a nonlocal prior improves the detection of multiple causal variants
Source: Bioinformatics. 2023 Jun 22;39(7):btad396. doi: 10.1093/bioinformatics/btad396 (PMC10326304; doi:10.1093/bioinformatics/btad396)
Supplement: btad396_Supplementary_Data [file btad396_supplementary_data.pdf]

Supplementary material: Genetic fine-mapping from summary data using a non-local prior improves detection of multiple causal variants

Ville Karhunen, Ilkka Launonen, Marjo-Riitta Järvelin, Sylvain Sebert, Mikko J Sillanpää

Contact info: ville.karhunen@oulu.fi

Contents

Supplementary Methods **4**

Theoretical considerations of non-local priors 4

Selecting a suitable value for τ based on sample size 4

Approximate Laplace's method 7

Clumping extremely highly correlated variants 8

Consistency check for out-of-sample LD reference 9

Credible sets 10

Simulation studies 11

References **14**

List of Figures

1 Prior distribution for model dimension with different values of u, with number of variants $P = 500$ and the maximum model dimension $K = 10$, on absolute scale (left panel) and on the log-scale (right panel). 17

2 Comparison of credible set coverage and power (mean and 95% confidence intervals) for FiniMOM and SuSiE as a function of sample size in the simulation studies (calculated over 100 simulation replicates across all simulated scenarios), with phenotypic variance explained set at 0.015. LD: linkage disequilibrium; FiniMOM: Fine-mapping using inverse-moment priors; SuSiE: sum of single effects. 18

3 Comparison of credible set coverage and power (mean and 95% confidence intervals) for FiniMOM and SuSiE with varying values for phenotypic variance explained (R^2) in the simulation studies (calculated over 100 simulation replicates across all simulated scenarios). LD: linkage disequilibrium; FiniMOM: Fine-mapping using inverse-moment priors; SuSiE: sum of single effects. 19

4 Comparison of credible set size (median and 95% confidence intervals) for FiniMOM and SuSiE in the simulation study in Northern Finland Birth Cohort 1966 (calculated over 100 simulation replicates). LD: linkage disequilibrium; FiniMOM: Fine-mapping using inverse-moment priors; SuSiE: sum of single effects. 19

5 Comparison of computing run times (median and 95% confidence intervals) for FiniMOM and SuSiE in the simulation study using synthetic genotype data (calculated over 100 simulation replicates across all simulated scenarios). The run times are based on a total of 12,500 Markov Chain Monte Carlo iterations for FiniMOM and a maximum of 100 iterations for SuSiE algorithm. FiniMOM: Fine-mapping using inverse-moment priors; SuSiE: sum of single effects. 20

6 Comparison of posterior inclusion probabilities (PIPs) and their ranks for FiniMOM and SuSiE in the simulation study in Northern Finland Birth Cohort 1966 (calculated over 100 simulation replicates across all simulated scenarios). Left panel: comparison of the PIPs for the causal variants. Right panel: comparison of the ranks of the causal variants (according to PIPs) on a logarithmic scale. The ranks were calculated by the descending order of all variants by their PIPs. In the case of ties, the mean value for the ranks was used. FiniMOM: Fine-mapping using inverse-moment priors; SuSiE: sum of single effects. 21

7 Comparison of the number of credible sets for FiniMOM and SuSiE in the simulation study in Northern Finland Birth Cohort 1966 (calculated over 100 simulation replicates across all simulated scenarios). The different panels correspond to the different numbers of simulated causal variants. FiniMOM: Fine-mapping using inverse-moment priors; SuSiE: sum of single effects. 22

8 Locus plot of genetic associations ($-\log_{10}(p)$) per each variant within *RAD17* locus (± 1Mb from rs17229943 variant), with credible sets highlighted for FiniMOM (left panels) and SuSiE (right panels) in the discovery GWAS (top panels) and in the replication GWAS (bottom panels). FiniMOM: fine-mapping using inverse-moment prior; SuSiE: sum of single effects; GWAS: genome-wide association study. 22

9 Locus plot of genetic associations ($-\log_{10}(p)$) per each variant within *BCO2* locus (± 1Mb from rs12420140 variant), with credible sets highlighted for FiniMOM (left panels) and SuSiE (right panels) in the discovery GWAS (top panels) and in the replication GWAS (bottom panels). FiniMOM: fine-mapping using inverse-moment prior; SuSiE: sum of single effects; GWAS: genome-wide association study. 23

10 Comparison of posterior inclusion probabilities (PIPs) for the variants in the credible sets within $NLRC4$ locus. Only variants in the credible sets are plotted. SuSiE credible set included 45 variants, while FiniMOM credible set included two variants. The variant rs385076 with the highest PIP for both methods is labelled in the figure. FiniMOM: Fine-mapping using inverse-moment priors; SuSiE: sum of single effects. 24

List of Tables

1 Values for τ in the simulation study using Northern Finland Birth Cohort 1966 genotype data, $N = 5400$. 6

2 Hyperparameters used in FiniMOM. 6

3 Genomic regions used in the simulations. 17

Supplementary Methods

Theoretical considerations of non-local priors

In Bayesian fine-mapping literature, the most common prior for the causal effects of individual variants is the Gaussian distribution (Hutchinson, Asimit, and Wallace 2020). Literature on Bayesian polygenic modelling (including prediction modelling) of quantitative traits have presented numerous other suggestions for the effect size distribution, such as t-distribution, double exponential, as well as their various mixtures (de los Campos et al. 2013; Zhou, Carbonetto, and Stephens 2013). Other propositions include the gamma distribution with different shape parameter values (Otto and Jones 2000; Xu 2003).

Our proposition is to use the product inverse-moment (piMOM) distribution as the prior for the causal effects. The prior originates from the inverse-moment priors presented in Johnson and Rossell (2010), where the authors highlight benefits of the non-local formulation of the prior particularly for variable selection. The core idea is to distinguish the estimated effects that strongly favor the 'alternative hypothesis', or conversely, the effects that distinctly differ from the parameter null value. In the scenarios of multiple causal variants per locus, piMOM achieves strong model selection due to the product formulation of the prior (Johnson and Rossell 2012). This is because if any of the effects is at zero, this shrinks the full density to zero. In comparison with the earlier literature, the piMOM prior bears the most resemblances to the gamma prior, as the functional form of the iMOM prior is related to the inverse-gamma distribution (Johnson and Rossell 2010).

It is worth highlighting that we are mainly interested in variable selection to identify the correct *causal configuration*. Therefore, the effect sizes themselves are not of interest, as they are integrated out in calculating the marginal likelihood for the data, given a specific model (main text equation (5)).

Selecting a suitable value for τ based on sample size

Let $\hat{z}_j = \hat{\beta}_j / \text{SE}(\hat{\beta}_j),\ j = 1, \ldots, P$. Assuming large sample size N, then under the null, approximately $\hat{z}_j \sim \mathcal{N}(0, 1)$, and if the genotype data have been standardized to mean zero and unit variance, then $\text{SE}(\hat{\beta}_j) \approx N^{-1/2}$, and

$$\hat{\beta}_j \sim \mathcal{N}(0, (N^{-1/2})^2).$$

This directly shows that the marginal distribution of each $\hat{\beta}_j$ depends on the sample size.

The inverse-moment (iMOM) prior for the marginal effect sizes β_j is given by

$$f(\beta_j) = \frac{\tau^{r/2}}{\Gamma(r/2)}|\beta_j|^{-(r+1)} \exp\left(-\frac{\tau}{\beta_j^2}\right),$$

where r regulates the tail behavior and τ controls the spread of the prior. Throughout, we set $r = 1$. The value of τ can be set such that the absolute values of the marginal effect sizes β_j are larger than a specific threshold value β_q with a given probability $1 - q$, that is,

$$\mathbb{P}(|\beta_j| > \beta_q) = 1 - q.$$

This can be solved using the known relationship between the iMOM prior and (inverse-)gamma distribution (Johnson and Rossell 2010). In the context of genetic analysis, Sanyal et al. (2019) suggest $\beta_q = 0.01$ and $q = 0.01$, yielding $\tau = 0.0083$. We propose to let τ be dependent on the sample size by setting $\beta_q = N^{-1/2}z_q$, leading to

$$\mathbb{P}(|\beta_j| > N^{-1/2}z_q) = 1 - q, \tag{S1}$$

where z_q is a specific quantile from a Gaussian distribution. This can be interpreted such that we assign a probability of $1 - q$ for a causal variant to have the observed $|\hat{z}|$ larger than z_q. Importantly, we have made explicit in equation (S1) that the detectable causal effect sizes β_j depend on the sample size, such that larger sample size allows for smaller effect sizes to be detected. In fact, similar ideas were implicitly suggested in Otto and Jones (2000).

Equivalently, we can use equation (S1) to assign a probability of $1 - q$ for a causal variant to have its observed p-value under a specific threshold α, with $z_q = \Phi^{-1}(1 - \alpha/2)$, where Φ denotes the cumulative Gaussian distribution function. As an example, $z_q = 3.89$ corresponds to $\alpha = 0.0001$. The different τ used in the simulation studies in this work are listed in Supplementary Table 1. All hyperparameters in our model and their considerations are presented in Supplementary Table 2.

Of note, the prior distribution of the detectable causal effects depends on the sample size, which is not ideal for accurate effect estimation. However, as the aim of our method is model selection, there is a trade-off to be made between the detection (presence or absence) and the interpretability (magnitude) of the effects. The original argument for using non-local prior distributions is their ability to distinguish the effects that strongly favour the "alternative hypothesis" (Johnson and Rossell 2010). In this spirit, we believe the distribution for

Supplementary Table 1: Values for τ in the simulation study using Northern Finland Birth Cohort 1966 genotype data, $N = 5400$.

τ	Interpretation
0.0083	$\mathbb{P}(\lvert\beta_j\rvert > 0.01) = 0.99$
0.00538	$\mathbb{P}(\lvert\beta_j\rvert > N^{-1/2} z_q) = 0.95$, where $z_q = 3.89$ (corresponds to $\alpha = 0.0001$)
0.00385	$\mathbb{P}(\lvert\beta_j\rvert > N^{-1/2} z_q) = 0.95$, where $z_q = 3.29$ (corresponds to $\alpha = 0.001$)
0.00320	$\mathbb{P}(\lvert\beta_j\rvert > N^{-1/2} z_q) = 0.95$, where $z_q = 3$ (corresponds to $\alpha = 0.0027$)

Supplementary Table 2: Hyperparameters used in FiniMOM.

**Hyperparameter**	**Role**	**Details**
r	Tail behavior of the non-local prior.	Set at $r = 1$ to provide Cauchy-like tails known to avoid over-shrinking strong signals.
τ	Spread of the non-local prior.	See Supplementary Methods for strategies in selection.
a	Parameter in the beta-binomial distribution for the model dimension.	Set at $a = 1$ to provide a right-skewed prior for the model dimension with large variance, to prioritize smaller models.
b	Parameter in the beta-binomial distribution for the model dimension.	Set at $P^u, u > 1$ (see next row).
u	Parameter to control the model dimension.	Controls the beta-binomial prior, with larger values of u providing stronger priors towards smaller dimensions.

the detectable causal effects should also take into account the distribution under the null, that is, distribution for the non-causal effects, which is dependent on the sample size. This approach is more explicit in various empirical Bayes approaches for multiple testing correction (Efron 2008; Muralidharan 2010). Our method is somewhat related to the empirical Bayes methods, with the distinction that the null distribution is derived analytically, rather than calculated based on the data. An explicit empirical Bayes approach would indeed be a possible alternative for selecting τ in our model.

Approximate Laplace's method

Consider the integral

$$\pi_{\boldsymbol{m}}(\boldsymbol{D}) = \int \pi(\hat{\boldsymbol{\beta}}|\boldsymbol{\beta}_{\boldsymbol{m}})\pi_{\boldsymbol{\beta}}(\boldsymbol{\beta}_{\boldsymbol{m}})d\boldsymbol{\beta}_{\boldsymbol{m}} = \int e^{-f(\boldsymbol{\beta}_{\boldsymbol{m}})}d\boldsymbol{\beta}_{\boldsymbol{m}}.$$

Throughout, we condition on model $\boldsymbol{m}$ of dimension d, and therefore we drop the subscript $\boldsymbol{m}$ from $\boldsymbol{\beta}_{\boldsymbol{m}}$ for simplicity. Assuming f is twice-differentiable, it can be approximated by a second-order Taylor expansion for $f(\boldsymbol{\beta})$ near $\tilde{\boldsymbol{\beta}}$ as

$$f(\boldsymbol{\beta}) \approx f(\tilde{\boldsymbol{\beta}}) + \boldsymbol{g}_{\tilde{\boldsymbol{\beta}}}(\boldsymbol{\beta} - \tilde{\boldsymbol{\beta}}) + \frac{1}{2}(\boldsymbol{\beta} - \tilde{\boldsymbol{\beta}})^T \boldsymbol{H}_{\tilde{\boldsymbol{\beta}}}(\boldsymbol{\beta} - \tilde{\boldsymbol{\beta}}), \tag{S2}$$

where $\boldsymbol{g}_{\boldsymbol{\beta}}$ and $\boldsymbol{H}_{\boldsymbol{\beta}}$ are the gradient and Hessian of f, respectively, evaluated at $\boldsymbol{\beta}$.

Laplace's method to approximate $\pi_{\boldsymbol{m}}(\boldsymbol{D})$ (Tierney and Kadane 1986) is based on equation (S2) at $\tilde{\boldsymbol{\beta}} = \text{argmin}_{\boldsymbol{\beta}} f$, where $\boldsymbol{g}_{\tilde{\boldsymbol{\beta}}} = 0$. Moreover, the value of the following Gaussian integral is known:

$$\int e^{-\frac{1}{2}(\boldsymbol{\beta}-\tilde{\boldsymbol{\beta}})^T H_{\tilde{\boldsymbol{\beta}}}(\boldsymbol{\beta}-\tilde{\boldsymbol{\beta}})}d\boldsymbol{\beta} = |2\pi \boldsymbol{H}_{\tilde{\boldsymbol{\beta}}}^{-1}|^{1/2}.$$

This leads to the following conventional *Laplace approximation* (Tierney and Kadane 1986; Johnson and Rossell 2012):

$$\pi_{\boldsymbol{m}}(\boldsymbol{D}) \approx e^{f(\tilde{\boldsymbol{\beta}})}(2\pi)^{d/2}|\boldsymbol{H}_{\tilde{\boldsymbol{\beta}}}|^{-1/2}. \tag{S3}$$

The value of $\tilde{\boldsymbol{\beta}}$ can be obtained using numerical optimization methods. While the Laplace's method switches the non-trivial integration problem to an optimization problem, the numerical optimization needs to be done at every iteration in our Markov Chain Monte Carlo algorithm, and therefore it is the main bottleneck in the computations.

As a rapid alternative, Rossell, Abril, and Bhattacharya (2021) proposed the approximate version of Laplace's method by evaluating the function f at a value where the gradient term does not vanish:

$$
\begin{aligned}
\pi_{\boldsymbol{m}}(\boldsymbol{D}) &\approx \int \exp\left\{-\left(f(\tilde{\boldsymbol{\beta}}) + \boldsymbol{g}_{\tilde{\boldsymbol{\beta}}}(\boldsymbol{\beta}-\tilde{\boldsymbol{\beta}}) + \frac{1}{2}(\boldsymbol{\beta}-\tilde{\boldsymbol{\beta}})^T \boldsymbol{H}_{\tilde{\boldsymbol{\beta}}}(\boldsymbol{\beta}-\tilde{\boldsymbol{\beta}})\right)\right\} d\boldsymbol{\beta} \\
&= \exp\{-f(\tilde{\boldsymbol{\beta}}) + \boldsymbol{g}_{\tilde{\boldsymbol{\beta}}}\tilde{\boldsymbol{\beta}} - \frac{1}{2}\tilde{\boldsymbol{\beta}}^T \boldsymbol{H}_{\tilde{\boldsymbol{\beta}}}\tilde{\boldsymbol{\beta}}\} \int \exp\{-\frac{1}{2}(\boldsymbol{\beta}^T \boldsymbol{H}_{\tilde{\boldsymbol{\beta}}}\boldsymbol{\beta} + 2\boldsymbol{\beta}(\boldsymbol{g}_{\boldsymbol{\beta}} - \tilde{\boldsymbol{\beta}}\boldsymbol{H}_{\tilde{\boldsymbol{\beta}}}))\} d\boldsymbol{\beta}.
\end{aligned}
$$

Denoting $\bar{\boldsymbol{\beta}} = \tilde{\boldsymbol{\beta}} - \boldsymbol{H}_{\tilde{\boldsymbol{\beta}}}^{-1}\boldsymbol{g}_{\tilde{\boldsymbol{\beta}}}$ and completing the square in the exponential in the integrand, we obtain

$$
\pi_{\boldsymbol{m}}(\boldsymbol{D}) \approx \exp\{-f(\tilde{\boldsymbol{\beta}}) + \boldsymbol{g}_{\tilde{\boldsymbol{\beta}}}\tilde{\boldsymbol{\beta}} - \frac{1}{2}\tilde{\boldsymbol{\beta}}^T \boldsymbol{H}_{\tilde{\boldsymbol{\beta}}}\tilde{\boldsymbol{\beta}} + \frac{1}{2}\bar{\boldsymbol{\beta}}^T \boldsymbol{H}_{\tilde{\boldsymbol{\beta}}}\bar{\boldsymbol{\beta}}\} \int \exp\{-\frac{1}{2}(\boldsymbol{\beta}-\bar{\boldsymbol{\beta}})^T \boldsymbol{H}_{\tilde{\boldsymbol{\beta}}}(\boldsymbol{\beta}-\bar{\boldsymbol{\beta}})\} d\boldsymbol{\beta}. \quad \text{(S4)}
$$

In (S4), the Gaussian integral is again known, and the exponential can be further simplified to obtain the *approximate Laplace approximation*:

$$
\pi_{\boldsymbol{m}}(\boldsymbol{D}) \approx \exp\{f(\tilde{\boldsymbol{\beta}}) + \frac{1}{2}\boldsymbol{g}_{\tilde{\boldsymbol{\beta}}}^T \boldsymbol{H}_{\tilde{\boldsymbol{\beta}}}^{-1}\boldsymbol{g}_{\tilde{\boldsymbol{\beta}}}\}(2\pi)^{d/2}|\boldsymbol{H}_{\tilde{\boldsymbol{\beta}}}|^{-1/2}. \quad \text{(S5)}
$$

Notably, equation (S5) does not involve the optimization step. We use $\tilde{\boldsymbol{\beta}} = \boldsymbol{R}_m^{-1}\hat{\boldsymbol{\beta}}_m$, where the subscript m refers to the indices of the proposed model $\boldsymbol{m}$. In the context of genetic analyses, both true causal effect sizes and the observed $\hat{\beta}_j$ are typically very small, so $\tilde{\boldsymbol{\beta}}$ can be considered to be in the neighborhood of $\boldsymbol{\beta}$.

It should be noted that the approximate Laplace's method requires both $\boldsymbol{H}_{\tilde{\boldsymbol{\beta}}}$ and $\boldsymbol{R}_m$ to be invertible. We also observed that for a near-singular $\boldsymbol{R}_m$, the value of $\tilde{\boldsymbol{\beta}}$ becomes unstable. Therefore, in cases where either $\boldsymbol{H}_{\tilde{\boldsymbol{\beta}}}$ or $\boldsymbol{R}_m$ are not invertible, or the maximum non-diagonal absolute value of $\boldsymbol{R}_m > 0.99$, the conventional Laplace's method in equation (S3) is applied. Importantly, equation (S3) does not involve calculating the inverse of $\hat{\boldsymbol{R}}$, and therefore our proposed method is flexible even in the case of non-invertible LD matrices (see also Zou et al. (2022)). For further considerations of the accuracy of the approximations, see Tierney and Kadane (1986) and Rossell, Abril, and Bhattacharya (2021) for the conventional Laplace's method and the approximate Laplace's method, respectively.

Clumping extremely highly correlated variants

Extremely highly correlated variants, such as those with pairwise $r^2 > 0.99$, cannot be easily distinguished by any fine-mapping method. Therefore, we have added an option to clump such variants to be treated as one 'cluster' in the posterior sampling, with the lead variant (i.e. the one with the lowest marginal p-value) representing this group of variants in the sampling, yielding a speed improvement as a by-product.

Consequently, any credible set that would include the lead variant from a cluster of clumped variants would also include all other variants from the corresponding cluster.

Consistency check for out-of-sample LD reference

When using out-of-sample LD reference, there is a possibility of inconsistencies in that the observed test statistic does not correspond to the estimated LD between the variants; Zou et al. (2022) give a toy example of such scenario of two variants, with $\hat{\boldsymbol{z}} = (6\ 7)^T$ and $\hat{\boldsymbol{R}} = \left(\begin{smallmatrix} 1 & 1 \\ 1 & 1 \end{smallmatrix}\right)$.

We present an option to distinguish such variants. Considering a cluster of variants in extremely high LD, we assume the variant C with the lowest p-value as the true causal variant. As per Hormozdiari et al. (2014), we assume that the observed test statistic for any non-causal variant N in this cluster depends solely on its correlation with C. The joint distribution of the marginal z scores for C and N is therefore a multivariate Gaussian distribution:

$$\begin{bmatrix} \hat{z}_C \\ \hat{z}_N \end{bmatrix} \sim \mathcal{N}\left(\begin{bmatrix} z_C \\ r z_C \end{bmatrix}, \begin{bmatrix} 1 & r \\ r & 1 \end{bmatrix} \right).$$

For testing equality of the observed and expected z-scores for a non-causal variant, we obtain a test statistic

$$X^2 = \frac{(\hat{z}_N - r\hat{z}_C)^2}{2(1 - r_T)} \sim \chi_1^2.$$

In the denominator, we approximate r with r_T, the threshold used to define extremely highly correlated variants. If the observed $X^2 > q_{\chi_1^2}$, the LD structure is not considered to approximate the z scores, and the variants are assigned to separate clusters. By default, we use $q_{\chi_1^2} = \mathbb{E}(X^2) \approx 0.45$. This provides additional robustness to the clumping and serves as a check for LD misspecification of tightly-linked variants. We highlight that none of the variants are excluded from fine-mapping, but simply treated as separate, non-clumped clusters in the sampling phase. In the toy example, our method would distinguish the misspecified LD structure and treat the variants separately. Similar to SuSiE (Zou et al. 2022), our method also strongly prefers the second variant to be the causal one.

Credible sets

We define a credible set at level α as a set of one or more variants that contain a true causal variant with probability larger than α. More than one credible set is allowed; the posterior distribution of model dimension d can be used as the posterior distribution for the number of credible sets.

Our credible set definition is similar to the one described in Wang et al. (2020), and we provide a similar toy example here. Consider 20 genetic variants $G_1, \ldots, G_{20}$, of which two variants, G_1 and G_2, are causal on a phenotype of interest. Additionally, $cor(G_1, G_{11}) = 1$ and $cor(G_2, G_{12}) = 1$. No statistical method can distinguish between G_1 and G_{11} (or between G_2 and G_{12}) which one is the true causal variant.

The conventional way to construct the credible set would be to consider a single set of variants that contain all causal variants with a specified probability (The Wellcome Trust Case Control Consortium et al. 2012; Hormozdiari et al. 2014). In the toy example, this definition would provide G_1, G_2, G_{11}, G_{12} as the credible set. An alternative way applied in our method is to explicitly recognize the two different signals of two credible sets: one of G_1 and G_{11}, and the other of G_2 and G_{12}. These sets imply that the scenarios for causal variants would be (1) G_1 and G_2, (2) G_1 and G_{12}, (3) G_2 and G_{11}, or (4) G_{11} and G_{12}. However, G_1 and G_{11} or G_2 and G_{12} cannot simultaneously be causal.

To illustrate the creation of the credible sets, consider the following posterior probabilities for the models (PPM): $PPM(G_1, G_2) = 0.27, PPM(G_{11}, G_2) = 0.26, PPM(G_1, G_{12}) = 0.23, PPM(G_{11}, G_{12}) = 0.23$, $PPM(G_1, G_3) = 0.005$ and $PPM(G_1, G_2, G_3) = 0.005$. The model with the highest PPM, denoted by $\boldsymbol{m}_{HPPM}$, is the one with G_1 and G_2. As it consists of two variants, two credible sets are created. To create a 95% credible set, we consider models in which variants can replace the ones in $\boldsymbol{m}_{HPPM}$ up to total posterior probability exceeding 0.95, conditioned on the number of variants in $\boldsymbol{m}_{HPPM}$. Now, G_1 can be replaced by G_{11} (in the model G_{11}, G_2), G_2 can be replaced by G_{12} (in the model G_1, G_{12}), and both can be replaced simultaneously (model G_{11}, G_{12}). This has accumulated the posterior probability, conditioned on two variants, to $(0.27 + 0.26 + 0.23 + 0.23)/(0.27 + 0.26 + 0.23 + 0.23 + 0.005) = 0.995$. Therefore, the credible sets would be (1) G_1 and G_{11}, and (2) G_2 and G_{12}. G_3 is not considered for the credible set due to the accumulated posterior probability already exceeding the desired credible set level of 0.95.

In summary, our proposed way of constructing credible sets takes two distinct types of uncertainties simultaneously into account: the number of signals (i.e. the number of credible sets), and the uncertainty in the signals (i.e. the particular variants in the specific sets). Of note, even though credible sets need to be constructed at a selected level α, we do not necessarily expect the nominal coverage (say, 95%), as the fine-mapped loci are not a random sample of loci which harbor causal variants, but only of those with the

largest effect sizes (Hutchinson, Watson, and Wallace 2020).

Simulation studies

Real genotype data

The genotype data used for simulations were obtained from two Finnish population-based pregnancy-birth cohorts, Northern Finland Birth Cohort 1966 (NFBC1966) (University of Oulu 2022a; Nordström et al. 2022; Sabatti et al. 2009) and Northern Finland Birth Cohort 1986 (NFBC1986) (University of Oulu 2022b; Järvelin, Hartikainen-Sorri, and Rantakallio 1993).

Genotyping and quality control for both datasets are detailed elsewhere (Kaakinen 2012; Karhunen et al. 2021). Briefly, genotyping was done using Illumina HumanCNV-370DUO Analysis BeadChip (Illumina, California, USA) for NFBC1966 and Illumina HumanOmniExpressExome-8v1.2 platform for NFBC1986. After sample quality control procedures and consent withdrawals, genomic data were available for 5400 and 3743 individuals in NFBC1966 and NFBC1986, respectively. Both datasets were Imputed to Haplotype Reference Consortium panel, and the autosomal, biallelic markers were filtered for minor allele frequency > 0.01, imputation quality $R^2 > 0.5$ and Hardy-Weinberg equilibrium p-value $> 10^{-12}$.

In the simulation studies, the genotype data from NFBC1966 were used for phenotype simulations to create the summary-level data, and genotype data from NFBC1986 for estimating the out-of-sample linkage disequilibrium (LD) matrix. These data come from the same geographical region with no overlapping individuals, therefore the different datasets provide ideal out-of-sample LD references for each other. Due to the larger sample size in NFBC1966, we used this study to simulate the phenotype and the summary data, and NFBC1986 for out-of-sample LD reference.

Using NFBC1966 genotype data, we simulated a quantitative trait under the model

$$Y = X\beta + \varepsilon,$$

with C causal variants explaining a total variance of ϕ in the phenotype, with $C \in \{1, 2, 5\}$ and $\phi \in \{0.015, 0.03\}$, as follows:

1. Select indices c for the causal variants uniformly from $\{1, \ldots, P\}$, with the constraint that the maximum correlation between the causal variants is 0.95.

2. Draw the effect sizes with the constraint that the power to detect all individual causal effect sizes was > 0.8 at significance level $= 0.05$:

a. Draw C effect sizes b_c from $\mathcal{N}(0, 1)$.

b. Scale the b_c to have the desired total variance explained by simulating an unscaled phenotype Y' with

$$Y' = X_c^T b_c + \varepsilon_{Y'}, \quad \varepsilon_{Y'} \sim \mathcal{N}(0, \mathrm{Var}(X_c^T b_c)(\frac{1}{R^2} - 1))$$

and then rescaling

$$\beta_c = b_c / \mathrm{SD}(Y')$$
$$Y = Y' / \mathrm{SD}(Y').$$

c. Return to (a) if any of β_c do not achieve the desired power.

The simulated phenotype was regressed separately on all variants (standardized to mean = 0 and variance = 1) within the locus assuming an additive model. The resulting summary statistics (i.e. association estimates and their standard errors) and an LD matrix were given as inputs to the considered fine-mapping methods.

Synthetic dataset

The main simulations using NFBC1966 genotype data are for parameters which are typical in GWASs on protein levels. However for complex traits, the heritabilities of individual loci can be considerably smaller, and therefore require larger sample sizes. Therefore, to examine the influence of lower heritability and larger sample size to the performance of our proposed method, we used synthetic HAPNEST genotype data created by Wharrie et al. (2022) in additional simulations. We also used the synthetic data to compare the speed of our method with that of SuSiE's as a function of the number of genetic variants considered in fine-mapping.

The synthetic data generation is based on a stochastic model that simulates genotypes from existing reference genomes while simultaneously taking genetic coalescence, recombination and mutation into account (Wharrie et al. 2022). The full synthetic dataset consists of over 6.8 million single-nucleotide polymorphisms of 1,008,000 individuals, categorised to six different ancestries, available at https://www.ebi.ac.uk/biostudies/studies/S-

BSST936. We extracted 50,000 observations of simulated African ancestry, and extracted their genotype data from five separate loci in chromosome 22 of 500, 1000, 2000, 5000 and 10,000 variants. The different heritabilities considered were 0.001 and 0.015, with 1, 2 or 5 causal variants. The simulations for the lowest heritability of 0.001 were conducted on synthetic data only as the sample size of 5400 in our real genotype data is far too small compared to a typical sample size in GWAS summary statistics for complex traits.

The simulation strategy for the synthetic dataset was exactly the same as for the NFBC1966 dataset. We calculated the reference LD for the synthetic data based on a separate sample of 10,000 HAPNEST observations of simulated African ancestry. To determine a suitable value for hyperparameter τ, we solved for $\mathbb{P}(|\beta_j| > N^{-1/2} z_q) = 1 - q$ with $z_q = 3.29$ and $q = 0.05$, based on the simulation results using NFBC1966 genotype data, leading to $\tau = 0.000416$.

References

de los Campos, Gustavo, John M Hickey, Ricardo Pong-Wong, Hans D Daetwyler, and Mario P L Calus. 2013. "Whole-Genome Regression and Prediction Methods Applied to Plant and Animal Breeding." *Genetics* 193 (2): 327–45. https://doi.org/10.1534/genetics.112.143313.

Efron, Bradley. 2008. "Microarrays, Empirical Bayes and the Two-Groups Model." *Statistical Science* 23 (1). https://doi.org/10.1214/07-STS236.

Hormozdiari, Farhad, Emrah Kostem, Eun Yong Kang, Bogdan Pasaniuc, and Eleazar Eskin. 2014. "Identifying Causal Variants at Loci with Multiple Signals of Association." *Genetics* 198 (2): 497–508. https://doi.org/10.1534/genetics.114.167908.

Hutchinson, Anna, Jennifer Asimit, and Chris Wallace. 2020. "Fine-Mapping Genetic Associations." *Human Molecular Genetics* 29 (R1): R81–88. https://doi.org/10.1093/hmg/ddaa148.

Hutchinson, Anna, Hope Watson, and Chris Wallace. 2020. "Improving the Coverage of Credible Sets in Bayesian Genetic Fine-Mapping." *PLOS Computational Biology* 16 (4): e1007829. https://doi.org/10.1371/journal.pcbi.1007829.

Järvelin, Marjo Riitta, Anna-Liisa Hartikainen-Sorri, and Paula Rantakallio. 1993. "Labour Induction Policy in Hospitals of Different Levels of Specialisation." *BJOG: An International Journal of Obstetrics & Gynaecology* 100 (4): 310–15. https://doi.org/10.1111/j.1471-0528.1993.tb12971.x.

Johnson, Valen E., and David Rossell. 2010. "On the Use of Non-Local Prior Densities in Bayesian Hypothesis Tests." *Journal of the Royal Statistical Society: Series B (Statistical Methodology)* 72 (2): 143–70. https://doi.org/10.1111/j.1467-9868.2009.00730.x.

———. 2012. "Bayesian Model Selection in High-Dimensional Settings." *Journal of the American Statistical Association* 107 (498): 649–60. https://doi.org/10.1080/01621459.2012.682536.

Kaakinen, Marika. 2012. *Genetic and Life Course Determinants of Cardiovascular Risk Factors: Structural Equation Modelling of Complex Relations.* Doctoral Thesis. Oulu: University of Oulu.

Karhunen, Ville, Tom A. Bond, Verena Zuber, Tuula Hurtig, Irma Moilanen, Marjo-Riitta Järvelin, Marina Evangelou, and Alina Rodriguez. 2021. "The Link Between Attention Deficit Hyperactivity Disorder (ADHD) Symptoms and Obesity-Related Traits: Genetic and Prenatal Explanations." *Translational Psychiatry* 11 (1): 455. https://doi.org/10.1038/s41398-021-01584-4.

Muralidharan, Omkar. 2010. "An Empirical Bayes Mixture Method for Effect Size and False Discovery Rate Estimation." *The Annals of Applied Statistics* 4 (1). https://doi.org/10.1214/09-AOAS276.

Nordström, Tanja, Jouko Miettunen, Juha Auvinen, Leena Ala-Mursula, Sirkka Keinänen-Kiukaanniemi, Juha Veijola, Marjo-Riitta Järvelin, Sylvain Sebert, and Minna Männikkö. 2022.

"Cohort Profile: 46 Years of Follow-up of the Northern Finland Birth Cohort 1966 (NFBC1966)." *International Journal of Epidemiology* 50 (6): 1786–1787j. https://doi.org/10.1093/ije/dyab109.

Otto, Sarah P, and Corbin D Jones. 2000. "Detecting the Undetected: Estimating the Total Number of Loci Underlying a Quantitative Trait." *Genetics* 156 (4): 2093–2107. https://doi.org/10.1093/genetics/156.4.2093.

Rossell, David, Oriol Abril, and Anirban Bhattacharya. 2021. "Approximate Laplace Approximations for Scalable Model Selection." *Journal of the Royal Statistical Society: Series B (Statistical Methodology)* 83 (4): 853–79. https://doi.org/10.1111/rssb.12466.

Sabatti, Chiara, Susan K Service, Anna-Liisa Hartikainen, Anneli Pouta, Samuli Ripatti, Jae Brodsky, Chris G Jones, et al. 2009. "Genome-Wide Association Analysis of Metabolic Traits in a Birth Cohort from a Founder Population." *Nature Genetics* 41 (1): 35–46. https://doi.org/10.1038/ng.271.

Sanyal, Nilotpal, Min-Tzu Lo, Karolina Kauppi, Srdjan Djurovic, Ole A Andreassen, Valen E Johnson, and Chi-Hua Chen. 2019. "GWASinlps: Non-Local Prior Based Iterative SNP Selection Tool for Genome-Wide Association Studies." *Bioinformatics* 35 (1): 1–11. https://doi.org/10.1093/bioinformatics/bty472.

The Wellcome Trust Case Control Consortium, Julian B Maller, Gilean McVean, Jake Byrnes, Damjan Vukcevic, Kimmo Palin, Zhan Su, et al. 2012. "Bayesian Refinement of Association Signals for 14 Loci in 3 Common Diseases." *Nature Genetics* 44 (12): 1294–1301. https://doi.org/10.1038/ng.2435.

Tierney, Luke, and Joseph B. Kadane. 1986. "Accurate Approximations for Posterior Moments and Marginal Densities." *Journal of the American Statistical Association* 81 (393): 82–86. https://doi.org/10.1080/01621459.1986.10478240.

University of Oulu. 2022a. "University of Oulu: Northern Finland Birth Cohort 1966." http://urn.fi/urn:nbn:fi:att:bc1e5408-980e-4a62-b899-43bec3755243.

———. 2022b. "University of Oulu: Northern Finland Birth Cohort 1986," http://urn.fi/urn:nbn:fi:att:f5c10eef-3d25-4bd0-beb8-f2d59df95b8e.

Wang, Gao, Abhishek Sarkar, Peter Carbonetto, and Matthew Stephens. 2020. "A Simple New Approach to Variable Selection in Regression, with Application to Genetic Fine Mapping." *Journal of the Royal Statistical Society: Series B (Statistical Methodology)* 82 (5): 1273–1300. https://doi.org/10.1111/rssb.12388.

Wharrie, Sophie, Zhiyu Yang, Vishnu Raj, Remo Monti, Rahul Gupta, Ying Wang, Alicia Martin, et al. 2022. "HAPNEST: Efficient, Large-Scale Generation and Evaluation of Synthetic Datasets

for Genotypes and Phenotypes." Preprint. Bioinformatics. https://doi.org/10.1101/2022.12.22.521552.

Xu, Shizhong. 2003. "Estimating Polygenic Effects Using Markers of the Entire Genome." *Genetics* 163 (2): 789–801. https://doi.org/10.1093/genetics/163.2.789.

Zhou, Xiang, Peter Carbonetto, and Matthew Stephens. 2013. "Polygenic Modeling with Bayesian Sparse Linear Mixed Models." *PLoS Genetics* 9 (2): e1003264. https://doi.org/10.1371/journal.pgen.1003264.

Zou, Yuxin, Peter Carbonetto, Gao Wang, and Matthew Stephens. 2022. "Fine-Mapping from Summary Data with the 'Sum of Single Effects' Model." *PLOS Genetics* 18 (7): e1010299. https://doi.org/10.1371/journal.pgen.1010299.

Supplementary Table 3: Genomic regions used in the simulations.

Gene	Chromosome	Start*	End*	Number of variants
UMPS	3	124349213	124564040	723
RPS14	5	149722753	149929319	487
GCNT2	6	10392456	10729601	840
CSNK1A1L	13	37577398	37779803	387
MDGA2	14	47211134	48243999	2996

* ±100 kb from each gene, given in hg19 coordinates.

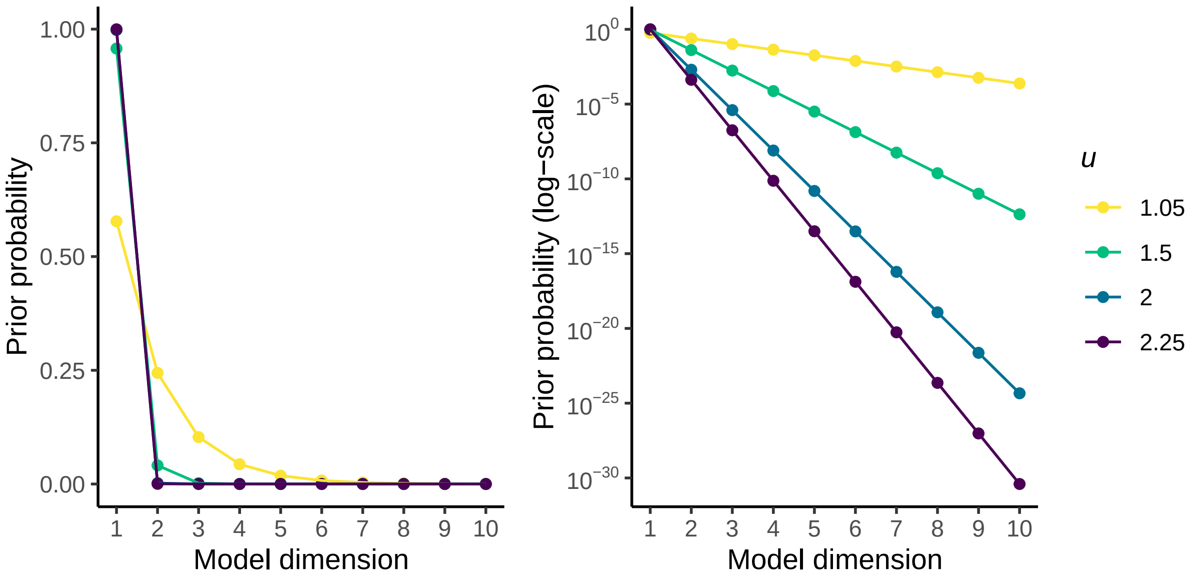

Supplementary Figure 1: Prior distribution for model dimension with different values of u, with number of variants $P = 500$ and the maximum model dimension $K = 10$, on absolute scale (left panel) and on the log-scale (right panel).

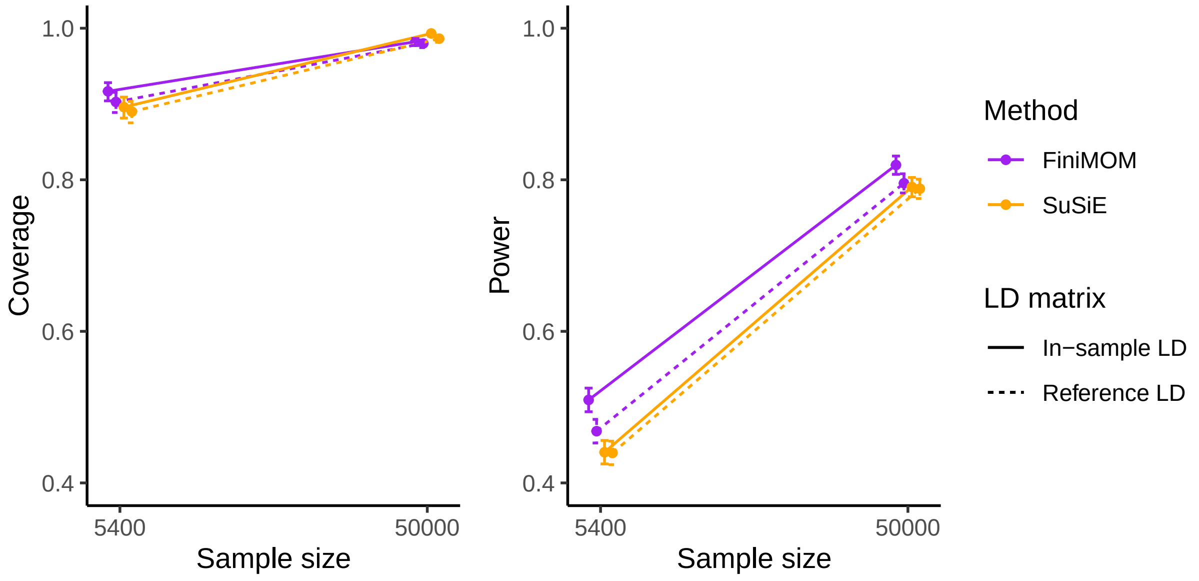

Supplementary Figure 2: Comparison of credible set coverage and power (mean and 95% confidence intervals) for FiniMOM and SuSiE as a function of sample size in the simulation studies (calculated over 100 simulation replicates across all simulated scenarios), with phenotypic variance explained set at 0.015. LD: linkage disequilibrium; FiniMOM: Fine-mapping using inverse-moment priors; SuSiE: sum of single effects.

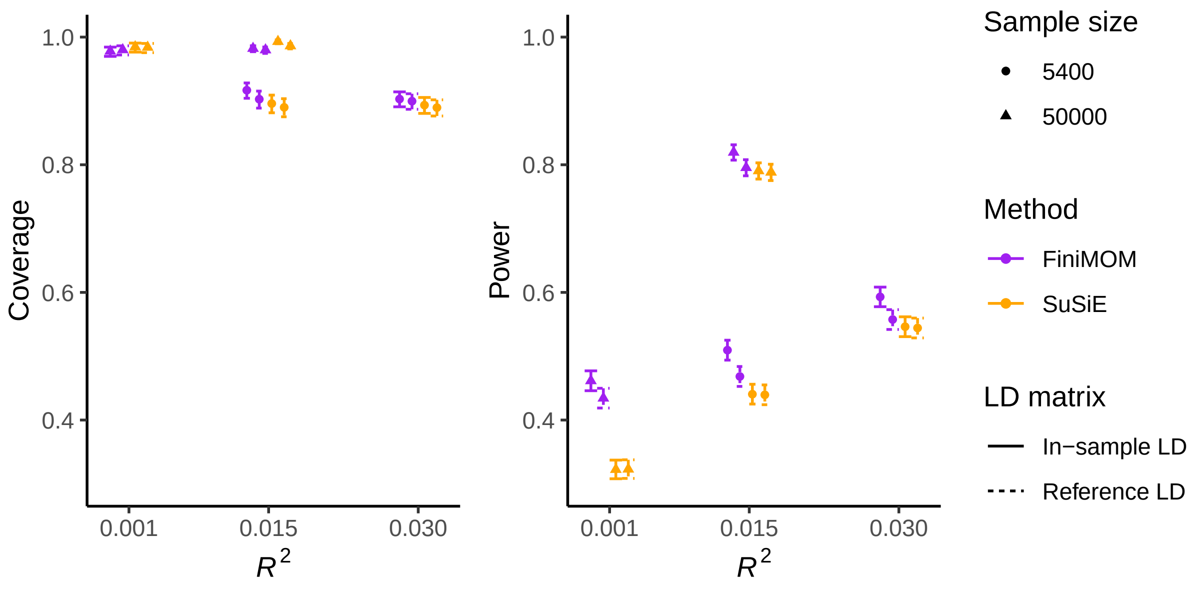

Supplementary Figure 3: Comparison of credible set coverage and power (mean and 95% confidence intervals) for FiniMOM and SuSiE with varying values for phenotypic variance explained (R^2) in the simulation studies (calculated over 100 simulation replicates across all simulated scenarios). LD: linkage disequilibrium; FiniMOM: Fine-mapping using inverse-moment priors; SuSiE: sum of single effects.

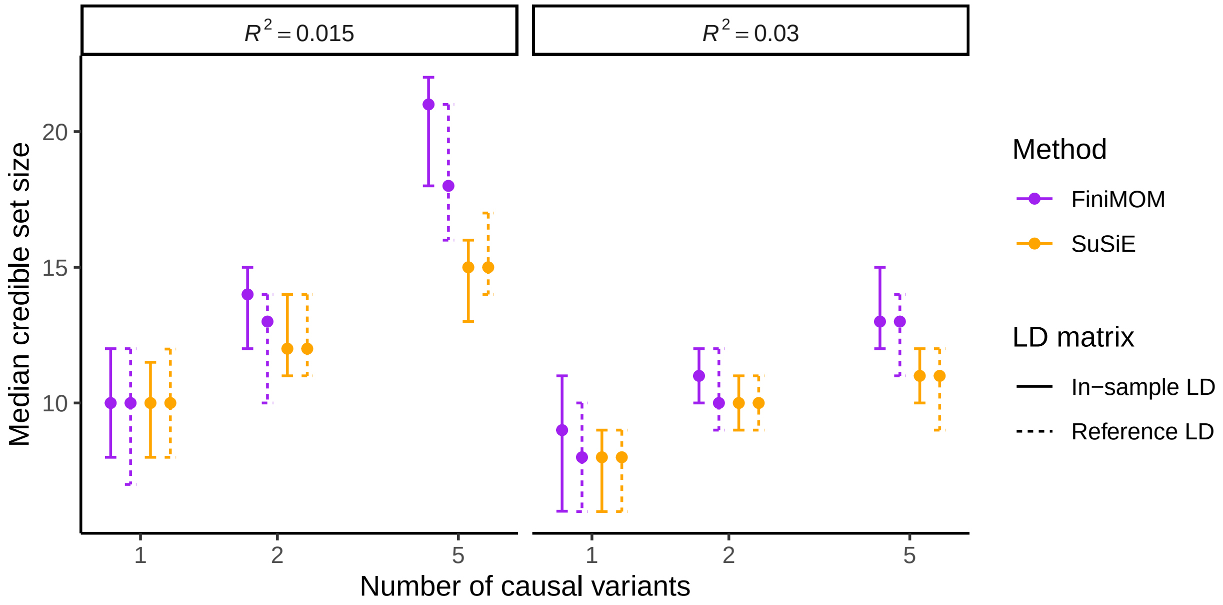

Supplementary Figure 4: Comparison of credible set size (median and 95% confidence intervals) for FiniMOM and SuSiE in the simulation study in Northern Finland Birth Cohort 1966 (calculated over 100 simulation replicates). LD: linkage disequilibrium; FiniMOM: Fine-mapping using inverse-moment priors; SuSiE: sum of single effects.

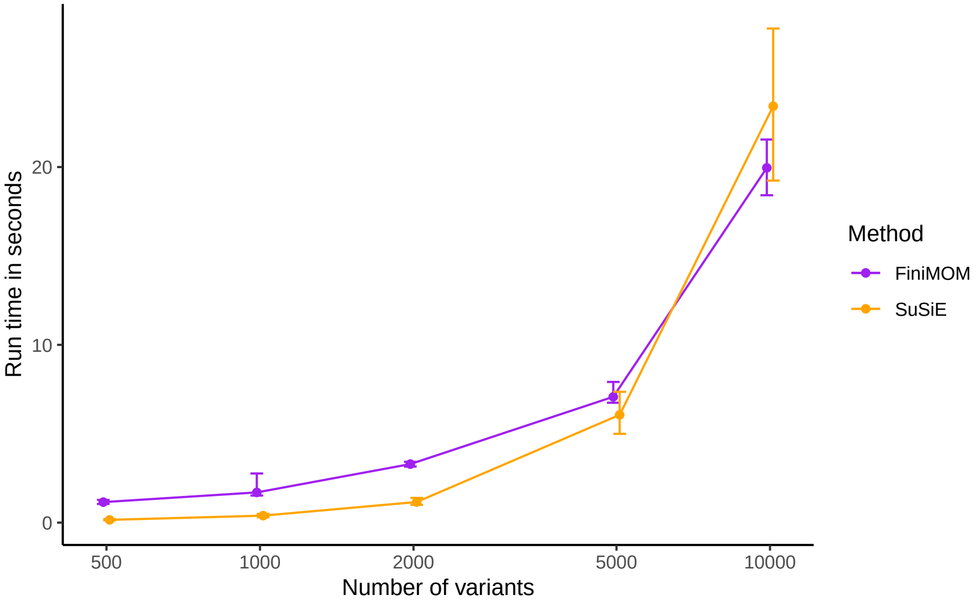

Supplementary Figure 5: Comparison of computing run times (median and 95% confidence intervals) for FiniMOM and SuSiE in the simulation study using synthetic genotype data (calculated over 100 simulation replicates across all simulated scenarios). The run times are based on a total of 12,500 Markov Chain Monte Carlo iterations for FiniMOM and a maximum of 100 iterations for SuSiE algorithm. FiniMOM: Fine-mapping using inverse-moment priors; SuSiE: sum of single effects.

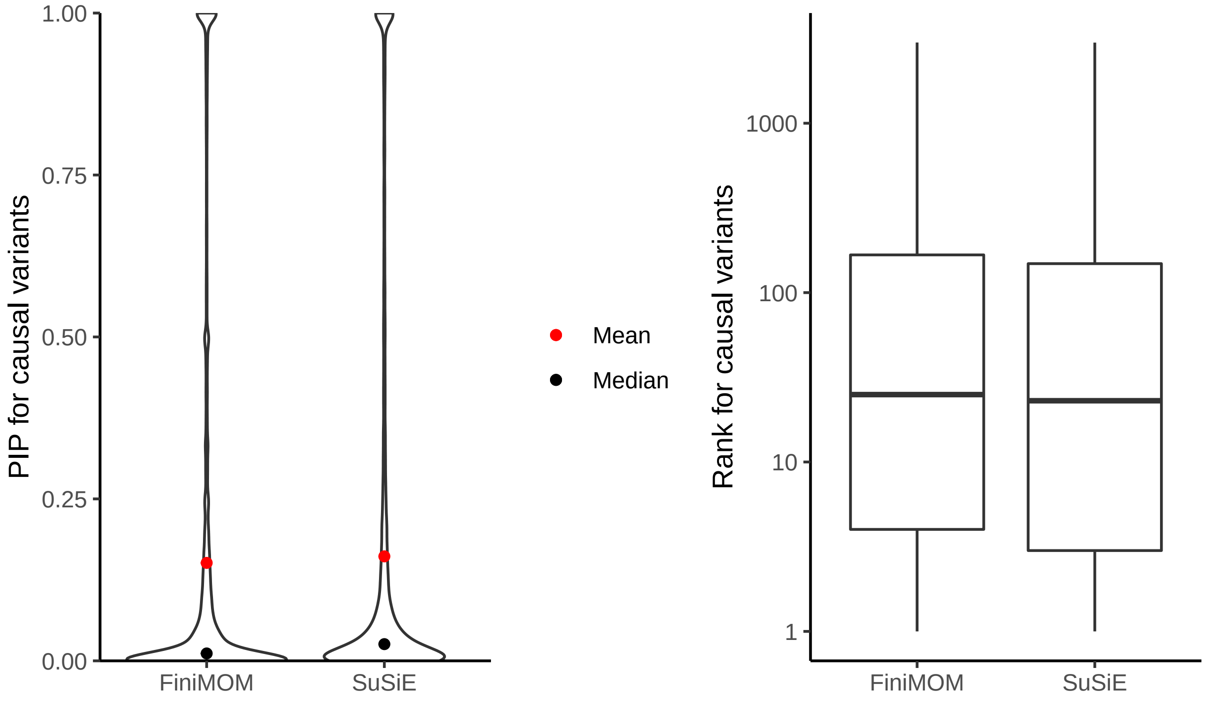

Supplementary Figure 6: Comparison of posterior inclusion probabilities (PIPs) and their ranks for FiniMOM and SuSiE in the simulation study in Northern Finland Birth Cohort 1966 (calculated over 100 simulation replicates across all simulated scenarios). Left panel: comparison of the PIPs for the causal variants. Right panel: comparison of the ranks of the causal variants (according to PIPs) on a logarithmic scale. The ranks were calculated by the descending order of all variants by their PIPs. In the case of ties, the mean value for the ranks was used. FiniMOM: Fine-mapping using inverse-moment priors; SuSiE: sum of single effects.

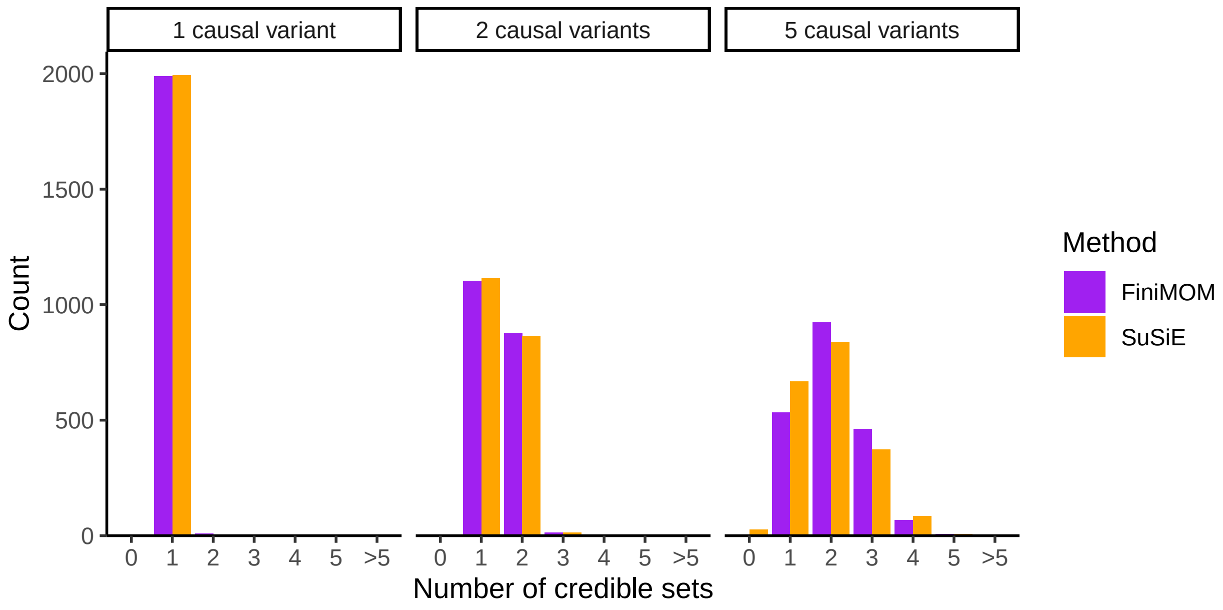

Supplementary Figure 7: Comparison of the number of credible sets for FiniMOM and SuSiE in the simulation study in Northern Finland Birth Cohort 1966 (calculated over 100 simulation replicates across all simulated scenarios). The different panels correspond to the different numbers of simulated causal variants. FiniMOM: Fine-mapping using inverse-moment priors; SuSiE: sum of single effects.

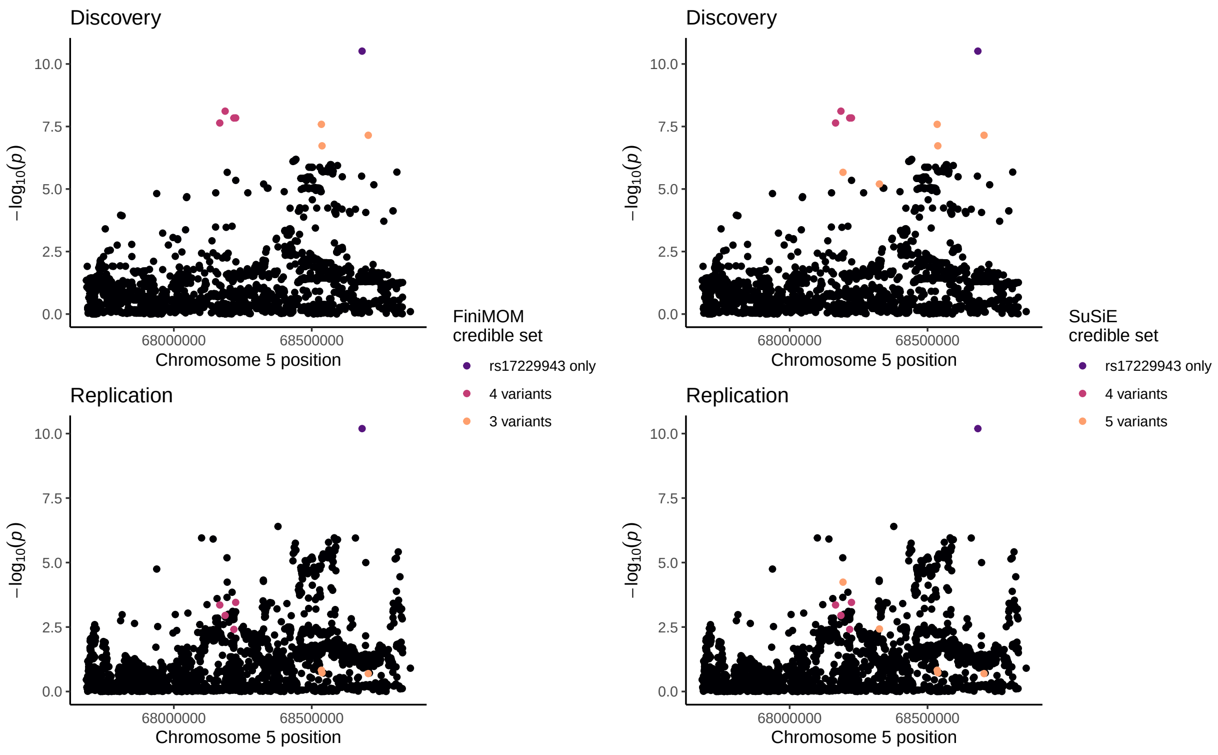

Supplementary Figure 8: Locus plot of genetic associations ($-\log_{10}(p)$) per each variant within *RAD17* locus ($\pm$ 1Mb from rs17229943 variant), with credible sets highlighted for FiniMOM (left panels) and SuSiE (right panels) in the discovery GWAS (top panels) and in the replication GWAS (bottom panels). FiniMOM: fine-mapping using inverse-moment prior; SuSiE: sum of single effects; GWAS: genome-wide association study.

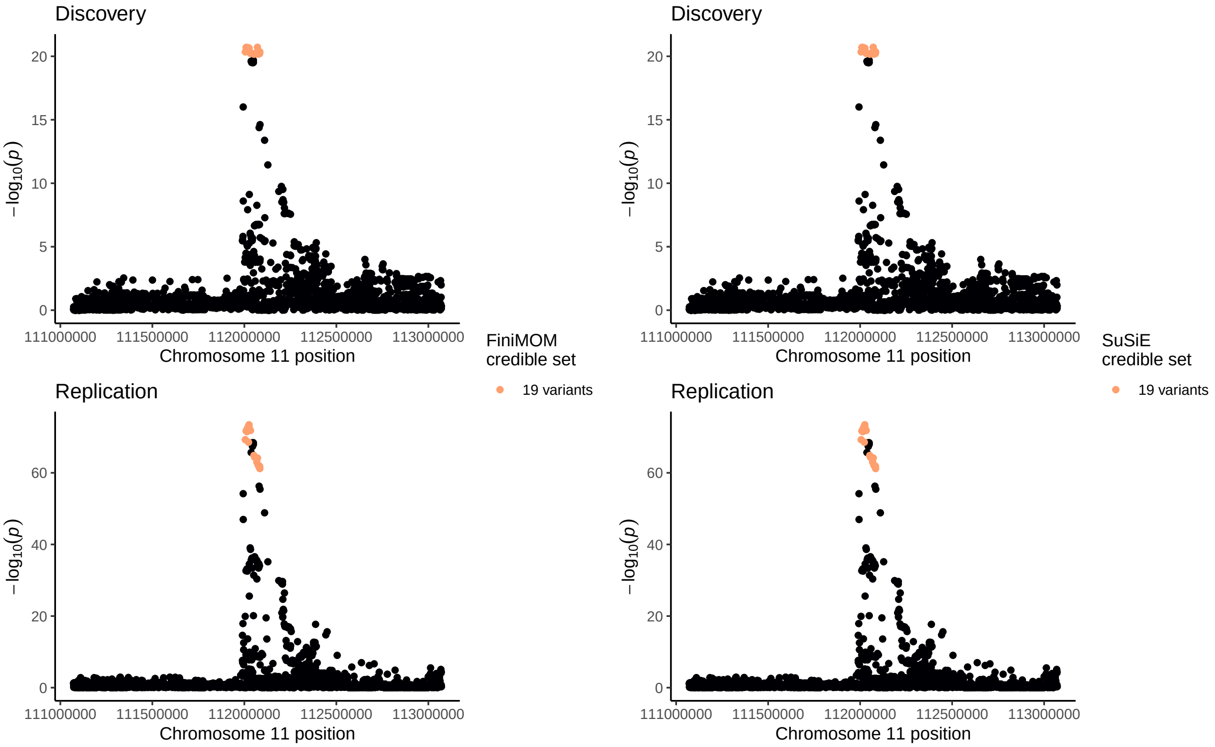

Supplementary Figure 9: Locus plot of genetic associations ($-\log_{10}(p)$) per each variant within *BCO2* locus ($\pm$ 1Mb from rs12420140 variant), with credible sets highlighted for FiniMOM (left panels) and SuSiE (right panels) in the discovery GWAS (top panels) and in the replication GWAS (bottom panels). FiniMOM: fine-mapping using inverse-moment prior; SuSiE: sum of single effects; GWAS: genome-wide association study.

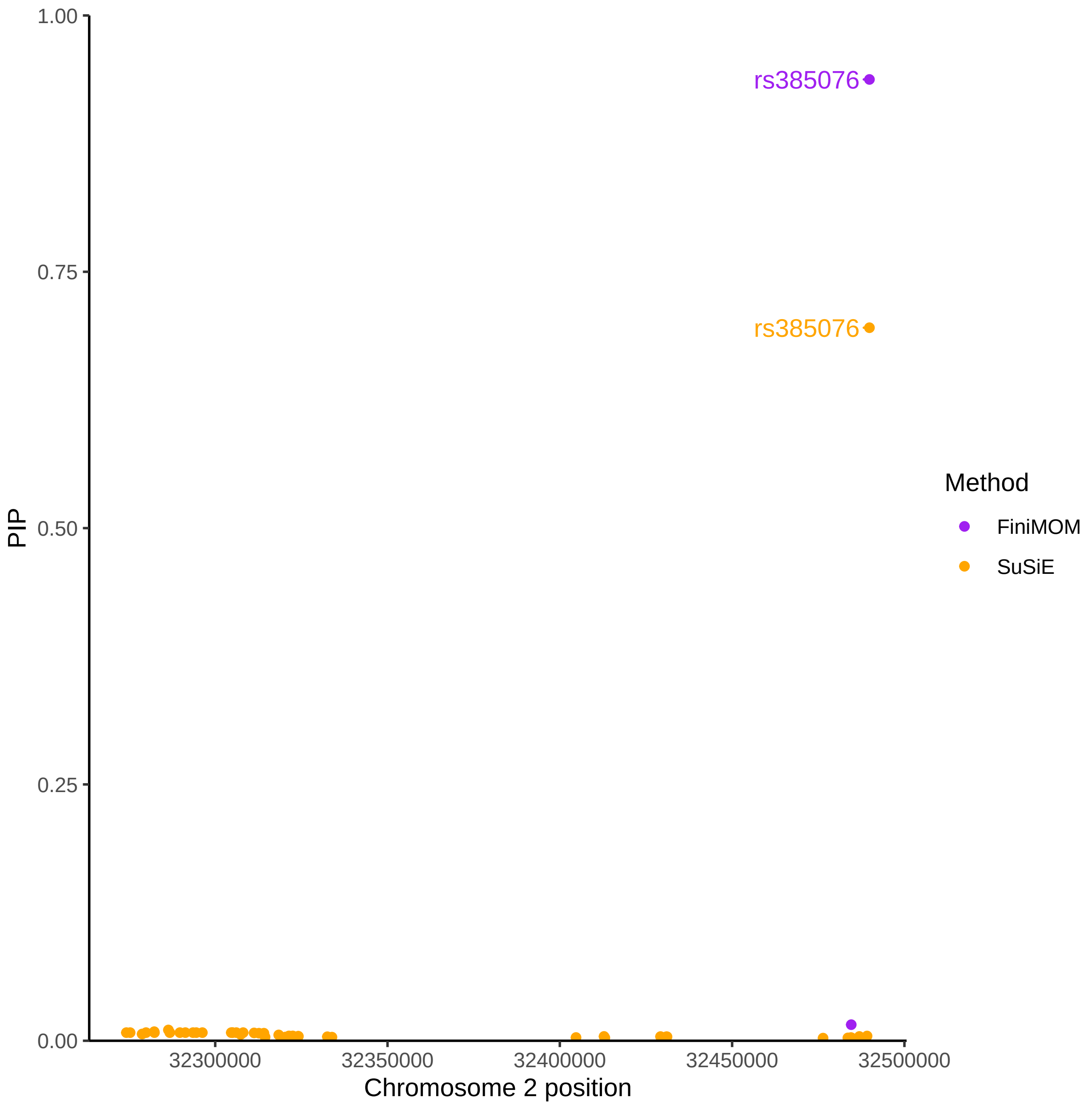

Supplementary Figure 10: Comparison of posterior inclusion probabilities (PIPs) for the variants in the credible sets within $NLRC4$ locus. Only variants in the credible sets are plotted. SuSiE credible set included 45 variants, while FiniMOM credible set included two variants. The variant rs385076 with the highest PIP for both methods is labelled in the figure. FiniMOM: Fine-mapping using inverse-moment priors; SuSiE: sum of single effects.
